# Chitosan induced cold tolerance in *Kobresia pygmaea* by regulating photosynthesis, antioxidant performance, and chloroplast ultrastructure

**DOI:** 10.3389/fpls.2024.1441564

**Published:** 2024-11-20

**Authors:** Shuo Li, Haoyang Sun, Ruolin Zhang, Cai Gao, Peizhi Yang, Xueqing He, Tianming Hu

**Affiliations:** College of Grassland Agriculture, Northwest A&F University, Yangling, Shaanxi, China

**Keywords:** *Kobresia pygmaea*, chitosan, cold stress, photosynthesis, antioxidant, chloroplast ultrastructure

## Abstract

**Introduction:**

Cold stress is the primary factor that limits the growth and development of *Kobresia pygmaea* in the Tibetan Plateau, China. Chitosan (CTS) has been recognized for its ability to enhance agricultural production and tolerance to stress.

**Methods:**

This study examined the effect of treating seedlings under cold stress with chitosan.

**Results and Discussion:**

The results demonstrated that cold stress inhibited the growth of seedlings and adversely affected the photosynthetic capacity [net photosynthetic rate (*Pn*), stomatal conductance (*Gs*), transpiration rate (*Tr*), maximum efficiency of photosystem II (*Fv/Fm*), quantum yield of photosystem II (φ*_PSII_*), electron transport rate (ETR), and non-light-induced non-photochemical fluorescence quenching Y(NPQ)] and destroyed PSII and the chloroplast structure. Under regular temperatures, low concentrations of CTS (0.005% and 0.01%) inhibited the soluble protein content, ribulose-1,5-bisphosphate carboxylase/oxygenase (Rubisco, EC 4.1.1.39) activity, and photosynthetic capacity. However, the application of 0.015% CTS increased the levels of soluble sugar, fructose, and protein, as well as those of the levels of ions, such as iron and magnesium, chlorophyll, photosynthetic capacity, and the activities of Rubisco, superoxide dismutase, and phenylalanine amino-lyase (PAL). Under cold stress, treatment with CTS decreased the contents of starch and sucrose; improved the contents of fructose, soluble protein, and antioxidants, such as ascorbic acid and glutathione; and enhanced the photosynthesis capacity and the activities of Rubisco, chitinase, and PAL. Exogenous CTS accelerated the development of the vascular bundle, mitigated the damage to chloroplast structure induced by cold, and promoted the formation of well-organized thylakoids and grana lamellae. Additionally, CTS upregulated the expression of genes related to cold tolerance in *K. pygmaea*, such as *KpBSK2*/*KpERF*/*KpDRE326*. These findings indicate that CTS enhances the cold tolerance in *K. pygmaea* by improving development of the vascular bundle, increasing the accumulation of solutes and antioxidants, regulating the transformation of carbohydrates, repairing the chloroplast structure, and maintaining the photosynthetic capacity and Rubisco activity.

## 1 Introduction

*Kobresia pygmaea* (C.B. Clarke) C.B. Clarke, Cyperaceae, is a dominant species of alpine meadows and covers more than 45,000 km^2^ of the Tibetan Plateau in China (Miehe et al., 2008, 2011). Taxonomic studies have shown that the genus *Kobresia* should be included within *Carex* and that *K. pygmaea* should be called *Carex parvula* O. Yano (C.B. Clarke) (Global Carex Group, 2015). Various environmental stresses in Tibet, such as rapid temperature shifts, exposure to intense ultraviolet radiation, and a low oxygen content, limit the growth of plants and the distribution of nutrients (Li et al., 2014). Cold conditions lead to the lipid peroxidation and the accumulation of malondialdehyde (MDA) in *K. pygmaea*, which triggers the production of soluble sugar in the seedlings to resist to cold stress (Xu et al., 2022a). *K. pygmaea* typically grows between 1 cm and 4 cm in areas fully exposed to sun without any protective cover. However, the mixed reproductive strategy of asexual and sexual reproduction of *K. pygmaea* endows it with strong vitality and competitiveness in its natural habitat. In grazing exclusion experiments, it can reach up to 20 cm when coverage by tree crowns or shaded by overgrown taller grasses (Miehe et al., 2019). Exposure of the soil to −2°C is lethal to *K. pygmaea* owing to the excessive respiration of the leaves over the photosynthesis rate (Gobel et al., 2019). In summary, previous studies have primarily concentrated on physiological changes across various temperatures. However, there is a dearth of research that explores efforts to increase the tolerance of plants to cold using agronomic measures.

Chitosan (CTS), a linear polysaccharide composed of β-1,4-glucosamines, is the deacylated form of chitin (Kumar, 2000). It has been described as an elicitor that activates the tolerance of plants to abiotic and biotic stress, such as NaCl, drought, cold, and plant fungal diseases (Jogaiah et al., 2020; Song et al., 2023; Yang et al., 2009; Zhou et al., 2018). As an elicitor, CTS stimulates the activities of chitinase and β-1,3-glucanase (β-1,3-GA), which have been shown to increase the resistance of garlic (*Allium sativum*) to *Fusarium* and promoted the activity of phenylalanine ammonia-lyase (PAL), the accumulation of phenolic compounds, and callose priming against fungal diseases at the infection site (Adamuchio-Oliveira et al., 2020; De Vega et al., 2021; Filyushin et al., 2022). Under drought stress, the exogenous application of CTS regulates the processes of antioxidant production, energy supply, and metabolic homeostatic processes, which are considered to be the key factors in improving the drought resistance of forage (Liu et al., 2020; Mustafa et al., 2022). CTS extended the storage time by enhancing ascorbate and the accumulation of total phenolics during postharvest cold storage (Castro-Cegrí et al., 2023; Elbagoury et al., 2022). Under cold stress, the foliar application of CTS also enhanced cold tolerance by regulating the process of photosynthesis and the production of antioxidants and osmotic compounds (Li et al., 2020; Tan et al., 2023; Zhou et al., 2018).

Cold stress destroys membrane fluidization, photosynthetic capacity, chloroplast structure, and normal functions of proteins, which inhibit the growth of plants (Cao et al., 2023; Guo et al., 2021). Moreover, cold stress impairs the structure and enzymatic functions of chloroplasts, which results in a reduction in the utilization of energy and a sudden increase in the production of reactive oxygen species (ROS) (Theocharis et al., 2012). Plants exposed to cold conditions promptly respond to cold signals by regulating various physiological mechanisms and expressing the genes that are associated with survival under cold stress, including the accumulation of osmoregulatory compounds, antioxidant defense systems (Hoermiller et al., 2022; Wang et al., 2024a), and plant hormones (Wang et al., 2024b), and the transport of signal molecules (Jiang et al., 2019). The cold acclimation confers cold tolerance on plants by triggering physiological and molecular mechanisms. In plants, the ROS scavenging system is activated to counteract oxidative damage through the action of antioxidant enzymes, including superoxide dismutase (SOD), peroxidase (POD), and ascorbate peroxidase (APX), and non-enzymatic antioxidants, such as ascorbic acid (AsA), glutathione (GSH), and phenolic and flavonoid compounds (Aslam et al., 2022; Mittler et al., 2022). Moreover, osmotic regulatory molecules, such as soluble sugars, soluble proteins, betaine, and proline, help to reduce osmotic pressure and stabilize the cell membranes to improve the tolerance of plants to cold stress (Gusain et al., 2023). A previous study indicated that warming *K. pygmaea* in open-top chambers (OTCs) improved their growth by increasing the accumulation of non-structural carbohydrates, including soluble sugars and starch, and activating the antioxidant systems to adapt to a warmer environment (Yang et al., 2012). In response to cold stress, plants undergo a multitude of molecular processes, including gene expression, in addition to exhibiting physiological changes (Browse and Xin, 2001). Transcription factors (TFs), including C repeat binding factor (CBF), CBF expression inducer 1 (ICE1), APETALA2/ethylene-responsive factor (AP2/ERF), and dehydration-responsive element binding (DREB1), are considered to be associated with the mechanism of cold tolerance (Waseem et al., 2024). CBF/DREB, a member of the AP2/EREBP family, is a key hub of the tolerance of plants to cold stress. The overexpression of *MbCBF1* in *Arabidopsis thaliana* enhanced tolerance to low temperatures (Liang et al., 2022). Studies on cucumber (*Cucumis sativus*) by Tan et al. (2023) showed that chitosan effectively enhanced the cold tolerance of these plants by promoting physiological changes, such as those of photosynthesis and antioxidant enzymes. Additionally, it was found that chitosan also affects transcriptomic responses. It primarily does this by affecting the pathways related to phenylalanine metabolism, plant hormone signal transduction, and MAPK signaling pathway—plant.

CTS, as a non-toxic and biodegradable molecular elicitor, is crucial to environmental safety and has been extensively utilized in stressed plants. Despite its recognized benefits, research on the potential of chitosan in modulating cold tolerance in alpine plants remains limited. This study examined the impact of chitosan on the cold stress response of *K. pygmaea*. We assessed the responses of seedlings to this elicitor, including plant morphological characteristics, osmoregulatory compounds, photosynthetic characteristics, mesophyll and chloroplast structure, antioxidant systems, including enzymes and antioxidants, and key genes associated with tolerance. These insights offer invaluable theoretical references for future research on the application of CTS to alpine plants.

## 2 Materials and methods

### 2.1 Plant materials and growth conditions

The seeds of *K. pygmaea* were collected from Damxung County, Tibet, China, in 2019, at an elevation of 4,200 m. The climatic conditions of this region include an average annual temperature of 1.3°C, average annual precipitation of 457 mm, and average annual sunshine duration of 2,880 h. The light period is 14 h/10 h from June to August, which would be the primary growth period. The seeds were stored at 4°C. Mature seeds were selected and disinfected sequentially with 75% ethanol and 1% sodium hypochlorite before they were utilized in experiments. The seeds were then subjected to 1 mol L^−1^ NaOH for 1 h to enhance their germination rate by disrupting the seed–coat barrier. This treatment resulted in a germination rate of 60%. The germinated seeds were cultivated in pots with vermiculite for more than 8 months to enable the formation of reproductive rhizomes. Seedlings propagated from rhizomes with emerging reproductive sites were then transplanted into 7×7 cm pots that contained sterilized vermiculite and placed in a controlled environmental chamber. The chamber was set to a relative humidity of 70% with a 14 h/10 h light/dark photoperiod at temperatures of 24°C/22°C and illuminated with cool white fluorescent lamps at a photosynthetic photon flux density of 400 μmol m^−2^ s^−1^. The seedlings were irrigated every 2 days with modified Hoagland’s nutrient solution (pH=6.5) (Fu et al., 2019).

### 2.2 Experimental material and treatments

When the seedlings had grown to approximately 20 cm high, the plants were sprayed with foliar CTS at concentrations of 0, 0.005%, 0.01%, 0.015%, and 0.02% (w/v) once every 2 days at 16:00–17:00. Chitosan with a deacetylation degree exceeding 95% (CAS: 9012-76-4) was purchased from Shanghai Macklin Biochemical Co. (Shanghai, China). Each solution contained 0.02% Tween 20 as a surfactant. After 7 days of pre-treatment with distilled water and chitosan, the seedlings were divided and transferred to artificial climate chambers set at two distinct temperatures: regular temperature (RT, 24°C) and low temperature (LT, 4°C). All the other cultivation parameters were maintained as previous settings. CTS was applied every 2 days during the 14 days of cold treatment. The characteristics of the photosynthetic and chlorophyll fluorescence were measured, and the ultrastructure of chloroplasts was observed by sectioning. Fully expanded fresh leaves from the plant center were sampled, immediately frozen in liquid nitrogen, and stored at −80°C for subsequent assays.

### 2.3 Assay of morphological, soluble substances, and near-infrared analysis

The height of plants was measured at each single third leaf from the center using a ruler. The leaf area (LA, cm^2^) was quantified from photographs using ImageJ (NIH, Bethesda, MD, USA). The leaves were then oven-dried at 65°C for 72 h to a constant weight to determine their dry biomass. The specific leaf area (SLA) was calculated as the ratio of leaf area to dry weight. The individual seedling was harvested, and the fresh weight was determined. They were then oven-dried to ascertain their dry weight. The dried seedlings were ground into powder and scanned using Near-Infrared Analysis (DA 7250, Perten Instruments, Segeltorp, Sweden) to determine the relative contents of sugar, calcium, crude protein, and cellulose dry matter among the different groups (Garcia and Cozzolino, 2006). The digested dry samples were analyzed for their contents of potassium (K), magnesium (Mg), and iron (Fe) using H_2_SO_4_–H_2_O_2_ as described by Sun et al. (2023). Furthermore, the contents of total nitrogen (N) and total phosphorus (P) were determined utilizing a San++ Compact Continuous Flow analyzer (Skalar Analytical B. V, Netherlands) as described by Liu G et al. (2022).

The content of total soluble proteins was quantified using the Bradford assay (Bradford, 1976). Fresh leaves of *K. pygmaea* (0.1 g) were homogenized in sodium phosphate buffer (50 mmol L^−1^, pH=7.0). The absorbance at 595 nm was measured based on the Coomassie brilliant blue (G-250) color development method. The concentration of total soluble protein was calculated from a standard curve using various concentrations of bovine serum albumin (BSA) and expressed as mg g^−1^ (FW).

The total soluble sugar and starch were quantified using the anthrone colorimetry method (Patel and Parida, 2021). Fresh leaves were treated with 80% ethanol and heated for 30 min, followed by extraction of the supernatant, steam drying, and reconstitution in distilled water. The anthrone–H_2_SO_4_ reagent was added to the solution, which was then incubated at 100°C for 10 min. Once the solution had cooled to ambient temperature, the A_620_ was measured using a U3900 spectrophotometer (Hitachi, Tokyo, Japan). The starch was quantified from the residue after extraction. The levels of absorbance of fructose and sucrose were measured at 480 nm using the resorcinol method (Foreman et al., 1973).

### 2.4 Determination of pigments content and Rubisco activity

The photosynthetic pigments were quantified as described by Sun et al. (2023). Fresh leaf samples (0.1 g) were extracted with 96% ethanol (v/v) in the dark for 72 h at room temperature. Their absorbance of supernatant was measured using a UV-VIS spectrophotometer at 665 nm, 649 nm, and 470 nm to quantify the chlorophyll *a* (Chl *a*), chlorophyll *b* (Chl *b*), and carotenoids (*Car*), respectively. The contents of pigments were calculated using the equations provided by Lichtenthaler and Wellburn (1983).

Rubisco was assayed using a kit (BC0440; Solarbio, Beijing, China).

### 2.5 Gas exchange and chlorophyll fluorescence analysis

The gas exchange parameters were assessed using a portable photosynthesis system (model LI-6400XT; Li-COR, Lincoln, NE, USA) (Zhang Y et al., 2021). The gas exchange indices were determined from the fresh leaves (the third and fourth leaves from center) at 9:00–11:00 after 14 days of cold treatment. A standard leaf chamber (2 ×3 cm^2^) fitted on a portable photosynthesis system was used at ambient relative humidity, 50–60%; carbon dioxide (CO_2_), 400 µmol mol^−1^; flowrate, 500 µmol s^−1^; vapor pressure deficit, 2; and photosynthetically active radiation, 1,200 µmol m^−1^ s^−1^. Each measurement was taken in different artificial climate chambers with different temperatures and sufficient time for equilibration in the chamber until constant readings were obtained. The indices that were measured included the net photosynthetic rate (*Pn*), stomatal conductance (*Gs*), intercellular CO_2_ concentration (*Ci*), and transpiration rate (*Tr*). The instantaneous water use efficiency (*WUE*) was the ratio of the net CO_2_ assimilation rate to the transpiration rate (*Pn*/*Tr*, μmol CO_2_ mmol H_2_O^−1^).

A Dual-PAM-100 fluorometer (Walz, Effeltrich, Germany) was utilized to determine the maximum efficiency of photosystem II (PSII) (*Fv/Fm*), quantum yield of PSII (φ*_PSII_*), electron transport rate (ETR), and the quantum yield of light-induced and non-light-induced non-photochemical fluorescence quenching [Y(NO) and Y(NPQ)]. The plants were adapted in the dark for at least 30 min before they were measured (Guo et al., 2021).

### 2.6 Observation of mesophyll structure and chloroplast ultrastructure

The anatomy of differentially treated seedlings was assessed. The leaves were sectioned into 5 mm × 5 mm pieces and immersed in a solution that contained 40% formaldehyde, 70% ethanol, and acetic acid (5:90:5, v/v/v) for 24 h. The samples were then dehydrated using a series of graded ethanol solutions before they were embedded in paraffin and sectioned with a microtome (Leica RM2016; Leica, Wetzlar, Germany). The sections were stained with Safranine and Fast green as described by Xu et al. (2022b) and imaged under a microscope (NIKON ECLIPSE E100; Nikon, Tokyo, Japan). The anatomical features of the leaf vein mechanical tissue area (μm^2^), vascular bundle area (μm^2^), xylem area (μm^2^), and phloem area (μm^2^) were measured using ImageJ.

Fresh leaves from each treatment were cut into pieces that measured approximately 1 mm^2^ and fixed in 4% glutaraldehyde at 4°C for 24 h. They were then rinsed in PBS and postfixed with osmium acid for 5 h. After dehydration in an ethanol series (30%, 50%, 70%, 80%, 90%, and 100% ethanol), the samples were embedded in Epon812 epoxy resin and sectioned using an ultramicrotome (Leica EMUC7; Leica, Austria). Ultrathin sections (70 nm) were double stained with uranyl acetate and lead citrate for 15 min. The sections were examined with a transmission electron microscope (TEM) (Hitachi HT7800, Japan).

### 2.7 The assay of ROS level and antioxidant characteristic

The content of malondialdehyde (MDA) was determined using the thiobarbituric acid method (Zhou et al., 2018). The production of superoxide radical (O_2_•^−^) was determined at 530 nm as described by Long et al. (2022).

Fresh leaf tissue (0.1 g) was homogenized with 50 mmol L^−1^ sodium phosphate buffer (pH=7.8) that contained 1% (w/v) polyvinylpolypyrrolidone (PVPP) to extract the enzymes. The centrifuged supernatants were used to assay SOD and POD as described by Wang et al. (2022).

*Determinations of antioxidant compounds*. Ascorbic acid (AsA) and dehydroascorbic acid (DHA) were performed using AsA and DHA Assay Kits, respectively (Cat. BC1230 and BC1240; Solarbio). The content of glutathione (GSH) was determined using 5,5′-dithiobis (2-nitrobenzoic acid) (DTNB), which produces a yellow product with a maximum absorbance at 412 nm, and is expressed as μg g^−1^ FW (Wang et al., 2022).

### 2.8 Assay of chitinase, β-1,3-GA, and PAL activity

The chitinase was assayed using a Chitinase Assay Kit (Cat. BC0820; Solarbio) according to the manufacturer’s instructions, which entailed measuring the absorbance at 585 nm in a Multi-detection Microplate Reader (Omega BioTek, Norcross, GA, USA) with 96-well microplates. The enzyme activity that was defined as the amount that catalyzed the production of 1 μg N-acetylglucosamine per hour was calculated from a standard curve and expressed as U g^−1^ FW.

The β-1,3-GA was assayed using a β-1,3-GA Assay Kit (Cat. BC0365; Solarbio) according to the manufacturer’s instructions. Its absorbance was measured at 540 nm with a Multi-detection Microplate Reader (Omega BioTek) with 96-well microplates and expressed as U g^−1^ FW.

L-Phenylalanine ammonia-lyase (PAL) was extracted as described by Christopoulos and Tsantili (2015). Fresh samples (0.2 g) were homogenized with 2 mL of 0.05 mol/L borate buffer (pH=8.8) that contained 5 mM β-mercaptoethanol (β-ME), 1 mmol L^−1^ EDTA-Na_2_, 5% glycerin (pH=8.3), and 5% (w/v) PVPP. The homogenates were centrifuged at 10,000×*g* for 15 min at 4°C. The supernatant was utilized as the crude enzyme extract. The PAL was assayed by measuring the amount of *trans*-cinnamic acid formed in the assay medium by spectrophotometry. One unit of PAL activity was defined as a 0.01 change at A_290_.

### 2.9 RNA extraction and RT-qPCR

The total RNA was isolated from the RT-CK, RT-0.015, LT-CK, and LT-0.015 samples using an RNA prep Pure kit (Vazyme, Dalian, China) and used to synthesize cDNA using a reverse transcriptase kit (Vazyme). Real-time quantitative reverse transcription PCR (qRT-PCR) was performed with the ChamQ SYBR qPCR Master Mix (Vazyme) using LightCycler480 II and the settings of Tian et al. (2024). The *K. littledalei KlGAPDH* (KAF3337947.1) gene served as the internal control gene (Sun et al., 2024). The primers for qRT-PCR were designed using Primer Premier v 5.0 (**Supplementary Table 1**) for the genes involved in cold response and tolerance, including *BSK2*, *Bam2*, *ERF*, *DRE326*, and the chitinase genes (*Chit197* and *Chit134*). The relative gene expression was calculate using the 2^−ΔΔCT^ method.

### 2.10 Statistical analysis

Statistical analyses were conducted using a one-way analysis of variance (ANOVA) and a Tukey’s range test using SPSS 20.0.0 (IBM, Inc., Armonk, NY, USA) at a threshold of *p* < 0.05. Each experiment had at least three biological replicates. The data are presented as the mean ± SD and plotted using Prism 8 software (GraphPad Software Inc., San Diego, CA, USA). A principal component analysis (PCA) and correlation analysis were performed using the Origin 2024 software (OriginLab, Northampton, MA, USA).

## 3 Results

### 3.1 Effect of chitosan on growth parameters and dry mass characteristic

**Figure 1:**
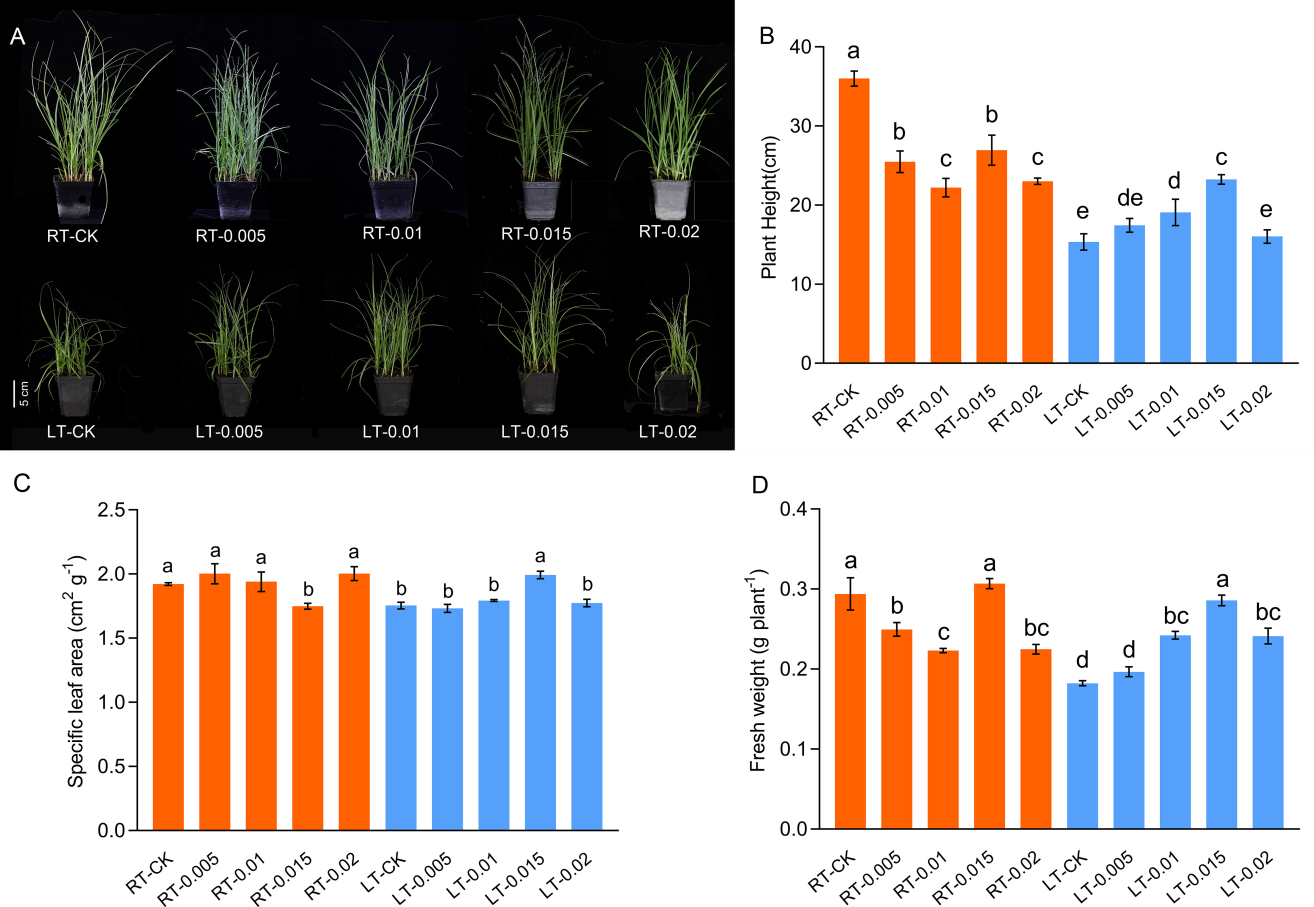
Effect of chitosan on the growth **(A)**, plant height **(B)**, specific leaf area **(C)**, and fresh weight **(D)** of *Kobresia pygmaea* seedlings. Data are represented as mean ± SD (n=3) of three biological replicates. Different letters above the vertical bars indicate significant differences at a *p* < 0.05 threshold according to Tukey’s range test.

The PCA analysis demonstrated the inter-relationship among the parameters, with distinct separations between the temperature treatments with and without the application of CTS shown in **Supplementary Figure 1**. Principal component 1 (PC1) and principal component 2 (PC2) explained 52.7% and 17.5% of the variance, respectively. In PC1, Y(NPQ), *Fv/Fm*, φ*_PSII_*, ETR, and APX were the predominant variables, whereas in PC2, soluble protein, *Tr*, *Gs*, fresh weight, and Rubisco and PAL activity were the predominant variables (**Supplementary Figure 1**). A two-way ANOVA indicated significant interactions between temperature and CTS concentration on the physiological parameters, including growth, gas exchange, chlorophyll fluorescence parameters, pigment levels, ROS, and specific defense enzymes and soluble substances (**Table 1**). There were significant differences in the height of plants associated with temperature and concentration (F-value = 129.30 and 10.62, respectively, *p* < 0.01), and specific leaf area (SLA) among the different temperatures (F-value=51.63). Cold stress led to a significant reduction in the height of *K. pygmaea* seedling height by 57.36% in the LT-CK group compared with the RT-CK group (*p*<0.05) and a decrease in the SLA and fresh weight by 8.74% and 37.97%, respectively (**Figure 1**). There was concentration effect, and the application of CTS at various concentrations ameliorated the growth of cold-stressed seedlings. The concentration of 0.015% was the most effective. It led to significant improvements in plant height, SLA, and fresh weight by 51.47%, 13.56%, and 56.83%, respectively, compared to the LT-CK group (**Figure 1**). However, the impact of CTS on the growth parameters was less pronounced under regular temperature conditions.

**Table 1:** F-value of ANOVA for growth parameters, chlorophyll content, gas exchange parameters, chlorophyll fluorescence parameters, photosynthetic enzymes, ROS characteristics, defense-related enzymes, solute substances, and forage quality indicators of *Kobresia pygmaea* under different temperature conditions with series of chitosan.

	Parameters	Temperature	Concentration	Temperature × Concentration
Growth parameters	Plant height	129.29**	10.62**	16.98**
	Specific leaf area	51.63**	1.03ns	33.50**
	Fresh weight	87.524**	67.424**	57.653**
Chlorophyll content	Tota-chl	267.66**	63.98**	37.17**
	Chla	296.23**	78.89**	65.34**
	Chlb	118.36**	24.20**	6.70**
	Car	373.07**	17.95**	11.46**
	Chl a/b	30.99**	5.57**	2.135ns
Gas exchange parameters	Pn	710.25**	115.90**	30.62**
	Gs	41.33**	24.21**	7.44**
	Ci	6.25*	29.47**	29.21**
	Tr	1.32ns	42.31**	25.94**
	WUE	203.89**	22.87**	56.67**
Chlorophyll fluorescence parameters	Fv/Fm	4,852.34**	29.39**	29.19**
	φPSII	5,848.03**	76.57**	45.13**
	ETR(II)	4,452.82**	165.26**	58.34**
	Y(NO)	12,592.53**	80.72**	77.00**
	Y(NPQ)	22,539.42**	206.74**	274.65**
Photosynthetic enzymes	Rubisco activity	174.24**	79.76**	28.88**
ROS characteristics	MDA	2.03ns	196.36**	36.04**
	O_2_^-^	315.43**	55.98**	17.99**
	POD	2,981.62**	203.40**	10.13**
	SOD	3,491.07**	323.99**	33.91**
	APX	44,820.87**	1212.00**	740.94**
	AsA	18,820.69**	369.53**	29.74**
	DHA	425.95**	150.66**	27.08**
	GSH	13,767.25**	90.46**	13.25**
Defense-related enzymes	Chitinase activity	3,554.04**	22.58**	265.84**
	β-1,3-GA activity	104.62**	7.26**	11.25**
	PAL activity	2,587.11**	622.52**	9.83**
Solute substances	Soluble sugar	262.85**	67.09**	14.35**
	Soluble protein	45.09**	33.84**	15.10**
	Fructose content	4610.96**	42.46**	3.96*
	Starch content	238.87**	193.28**	25.66**
	Sucrose	4,278.01**	94.47**	106.70**
	N content	501.51**	63.75**	33.66**
	P content	635.25**	219.61**	98.48**
	K content	423.65**	326.58**	120.27**
	Fe content	8.69**	48.64**	49.57**
	Mg content	416.32**	1262.27**	579.87**
Forage quality indicator (dry mass)	Sugar	86,087.81**	8233.52**	1958.1**
	Calcium	4,422.25**	373.594**	710.531**
	Crude protein	10.12**	1370.45**	810.50**
	Fiber	9,294.32**	43.67**	99.04**

*p < 0.05, **p < 0.01, ns, not significant (*p* ≥ 0.05).

A near-infrared analysis revealed preliminary characteristics of the dry samples, which indicated various physiological responses (**Supplementary Figure 3**). The plants that were cultivated at regular temperatures were associated with elevated contents of fiber and crude protein in the dry mass, whereas cold stress enhanced the contents of sugar and calcium in the dried samples. Moreover, the treatment of exogenous CTS augmented the accumulation of calcium and crude protein accumulation under cold stress, particularly in the LT-0.01 and LT-0.015 groups.

### 3.2 Effect of chitosan on soluble substance contents

**Figure 2:**
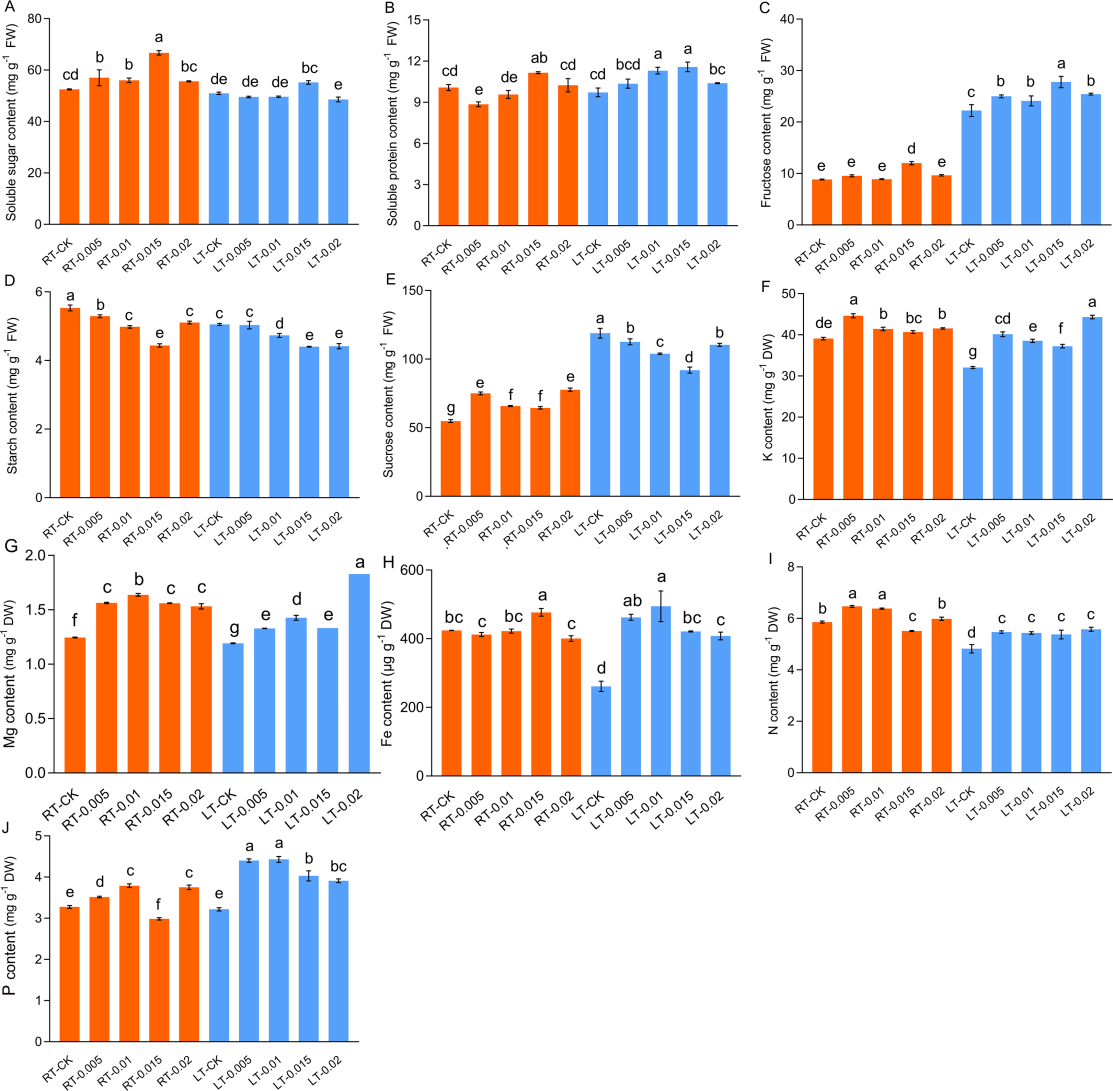
Effect of chitosan on the soluble sugar content **(A)**, soluble protein content **(B)**, fructose content **(C)**, starch content **(D)**, sucrose **(E)**, K content **(F)**, Mg content **(G)**, Fe content **(H)**, N content **(I)**, and P content **(J)** of *Kobresia pygmaea* leaves. Data are represented as mean ± SD (n=3). Different letters above the vertical bars indicate significant differences at a *p* < 0.05 threshold according to Tukey’s range test.

The contents of soluble protein, soluble sugar, and fructose increased in parallel with increasing concentrations of CTS, with optimal enhancement at 0.015%. Under both temperatures, the contents of soluble protein increased by 10.74% and 19.08%, respectively, following treatment with 0.015% CTS compared to the control groups (**Figure 2B**). Similarly, the contents of soluble sugar increased by 27.02% and 8.31%, respectively, and those of fructose increased by 35.92% and 24.95%, respectively (**Figures 2A, C**). In contrast, the contents of starch were diminished by cold stress and the application of CTS. Sucrose accumulated under cold stress, but this accumulation was mitigated by the application of exogenous CTS.

Cold stress significantly reduced the accumulation of K, Fe, and N, with no notable changes observed in the levels of Mg and total P. The application of exogenous CTS effectively mitigated these reductions and enhanced the levels of K, Mg, Fe, N, and P. In particular, under regular temperatures, 0.01% CTS was the optimal concentration to increase the contents of Mg, N, and P, while concentrations of 0.005% and 0.015% were the most effective for K and Fe, respectively (**Figure 2**).

### 3.3 Effect of chitosan on photosynthesis parameters

**Figure 3:**
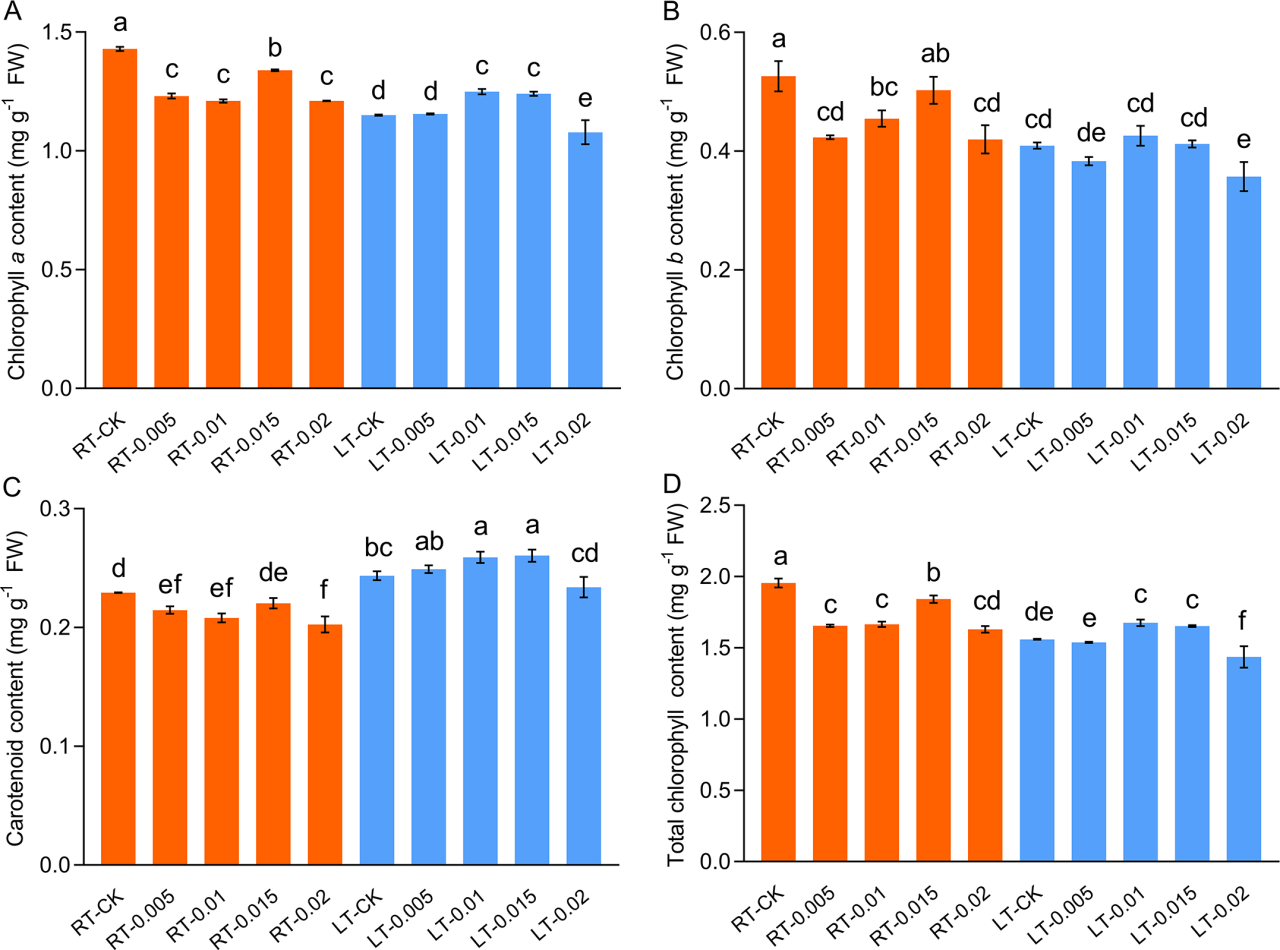
Effect of chitosan on the chlorophyll *a* content **(A)**, chlorophyll *b* content **(B)**, carotenoid content **(C)**, and total chlorophyll content **(D)** of *Kobresia pygmeae* leaves. Data are represented as mean ± SD (n=3). Different letters above the vertical bars indicate significant differences at a *p* < 0.05 threshold according to Tukey’s range test.

Cold stress inhibited the contents of photosynthetic pigments, including Chl *a* and Chl *b*. However, the levels of *Car* increased. The application of exogenous CTS at concentrations of 0.01% and 0.015% under cold stress resulted in an increase of 8.58% and 7.79% in the content of Chl *a*, respectively (**Figure 3**). Under regular temperatures, the contents of pigments initially decreased with the application of CTS and subsequently increased with higher concentrations, which reached a peak at 0.015% (w/v). This observation indicates that there is a regulatory effect of CTS on the accumulation of photosynthetic pigments that is dependent on concentration.

**Figure 4:**
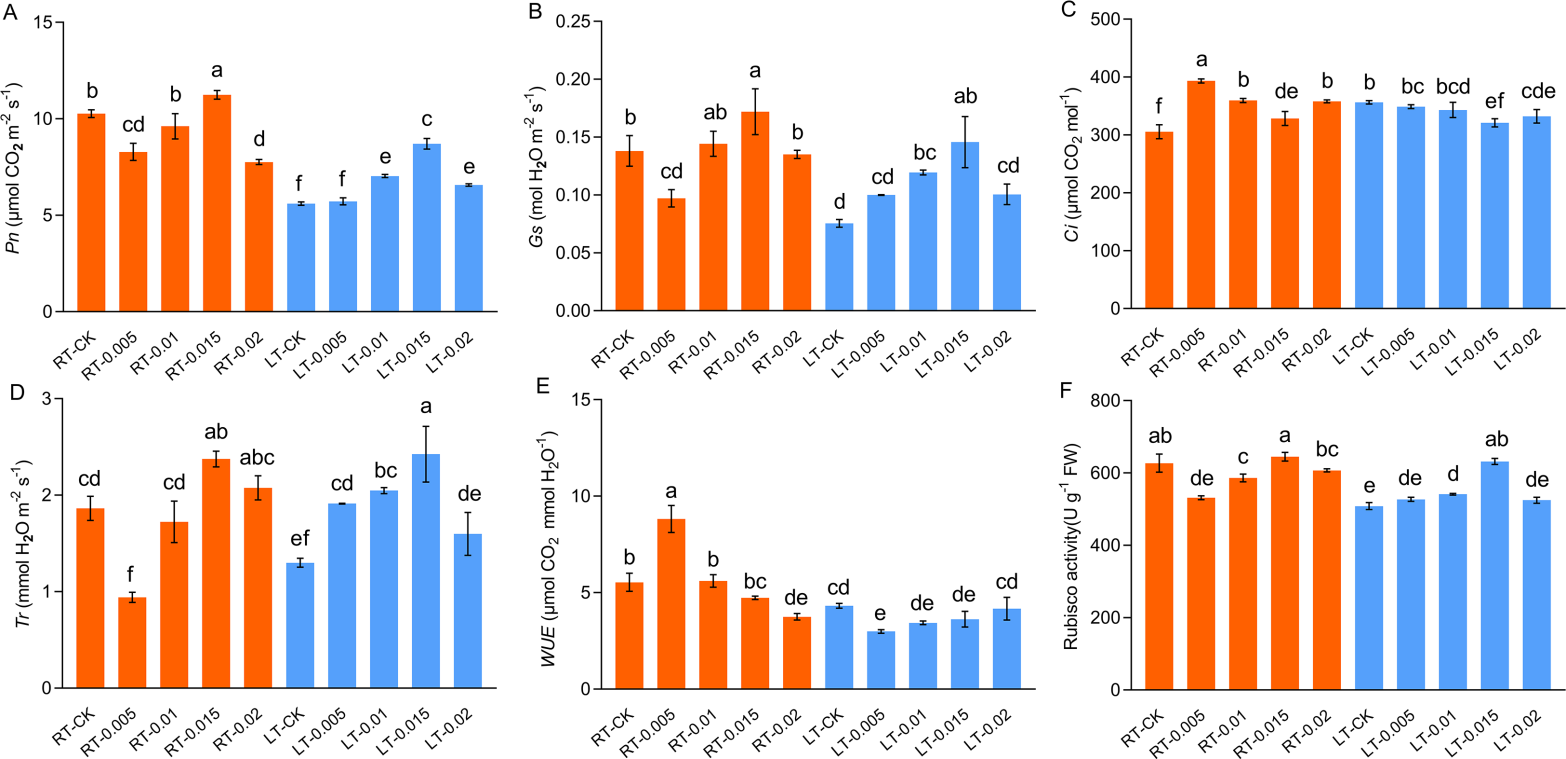
Effect of chitosan on the gas exchange parameters. *Pn*, net photosynthesis rate **(A)** *Gs*, stomatal conductance **(B)** *Ci*, intercellular CO_2_ **(C)** *Tr*, transpiration rate **(D)** *WUE*, instantaneous water use efficiency **(E)** Rubisco activity **(F)**. Data are represented as mean ± SD (n=4). Different letters above the vertical bars indicate significant differences at a *p* < 0.05 threshold according to Tukey’s range test.

Cold stress impaired the photosynthetic capacity of the *K. pygmaea* seedlings and inhibited their net photosynthetic rate (*Pn*), stomatal conductance (*Gs*), and transpiration rate (*Tr*), while elevating their intercellular CO_2_ concentration (*Ci*) (**Figure 4**). At regular temperatures, the treatment with exogenous CTS induced a significant 9.44% increase in the value of *Pn* in the RT-0.015 group (**Figure 4**). This aligned with the changes observed in the content of chlorophyll (**Figure 3**). The *Gs* and *Tr* were also enhanced by CTS, whereas the *Ci* decreased as the concentrations of CTS increased. Under cold stress, the photosynthetic parameters *Pn*, *Gs*, and *Tr* responded positively to the CTS treatment. These treatments optimally increased these parameters in the LT-0.015 group and achieved significant increases of 55.21%, 92.84%, and 86.67%, respectively, compared with the LT-CK group (**Figures 4A, B, D**). Collectively, these results demonstrated that exogenous CTS notably enhanced the photosynthetic capacity of *K. pygmaea* under cold stress.

The chlorophyll fluorescence parameters were examined to assess the impact of chitosan on the *K. pygmaea* seedlings. Cold stress diminished the fluorescence characteristics, such as *Fv/Fm*, φ*_PSII_*, ETR, and Y(NPQ), compared to the RT-CK group, with a significant increase in Y(NO) that indicated substantial photooxidative damage. Under regular temperature, the CTS had no significant effect on *Fv/Fm*, but it negatively regulated φ*_PSII_* and ETR with the exception of a concentration of 0.015% CTS (**Table 2**). Exogenous CTS enhanced the photochemical activity of PSII in the plants stressed by cold, with significant increases in *Fv/Fm*, φ*_PSII_*, ETR, and Y(NPQ) by 20.95%, 192.44%, 194.25%, and 55.84%, respectively, and a significant 18.16% decrease in Y(NO). These findings suggest that the application of CTS effectively ameliorates the detrimental effects of cold stress on photosynthetic performance of *K. pygmaea* seedlings.

**Table 2:** Effect of chitosan on the chlorophyll fluorescence indicators. Data are represented as mean ± SD (n=4).

Treatment	*Fv/Fm*	φ*_PSII_*	ETR	Y(NO)	Y(NPQ)
RT-CK	0.837 ± 0.012a	0.231 ± 0.008ab	82.725 ± 1.987a	0.229 ± 0.006f	0.584 ± 0.018a
RT-0.005	0.841 ± 0.005a	0.216 ± 0.003b	73.575 ± 4.366b	0.308 ± 0.008e	0.535 ± 0.002b
RT-0.01	0.838 ± 0.008a	0.215 ± 0.005b	75.875 ± 0.750b	0.300 ± 0.008e	0.569 ± 0.003a
RT-0.015	0.838 ± 0.003a	0.235 ± 0.016a	82.100 ± 3.732a	0.298 ± 0.002e	0.532 ± 0.005b
RT-0.02	0.834 ± 0.004a	0.218 ± 0.009b	56.375 ± 1.367c	0.346 ± 0.002d	0.541 ± 0.003b
LT-CK	0.549 ± 0.005e	0.043 ± 0.001e	16.525 ± 0.206f	0.800 ± 0.011a	0.205 ± 0.010f
LT-0.005	0.590 ± 0.011d	0.064 ± 0.003d	26.050 ± 1.103e	0.754 ± 0.001b	0.264 ± 0.008e
LT-0.01	0.626 ± 0.010c	0.064 ± 0.003d	25.025 ± 0.830e	0.732 ± 0.004b	0.292 ± 0.002d
LT-0.015	0.664 ± 0.018b	0.126 ± 0.001c	48.625 ± 0.320d	0.655 ± 0.032c	0.319 ± 0.004c
LT-0.02	0.599 ± 0.007d	0.043 ± 0.002e	17.000 ± 1.152f	0.825 ± 0.004a	0.157 ± 0.004g

Different letters indicate significant differences at a *p* < 0.05 threshold according to Tukey’s range test.

### 3.4 Effect of chitosan on Rubisco activity

Cold stress significantly inhibited the activity of Rubisco, which is consistent with the changes observed in *Pn* (**Figure 4**). At regular temperatures, the exogenous CTS suppressed the activity of Rubisco at low concentrations of CTS (0.005% and 0.01%), while it had no significant effect with spraying 0.015% CTS. Under cold stress, the 0.015% CTS treatment optimally ameliorated the Rubisco activity, which led to a notable 24.25% increase compared to the LT-CK group.

### 3.5 Effect of chitosan on mesophyll structure and chloroplast ultrastructure

**Figure 5:**
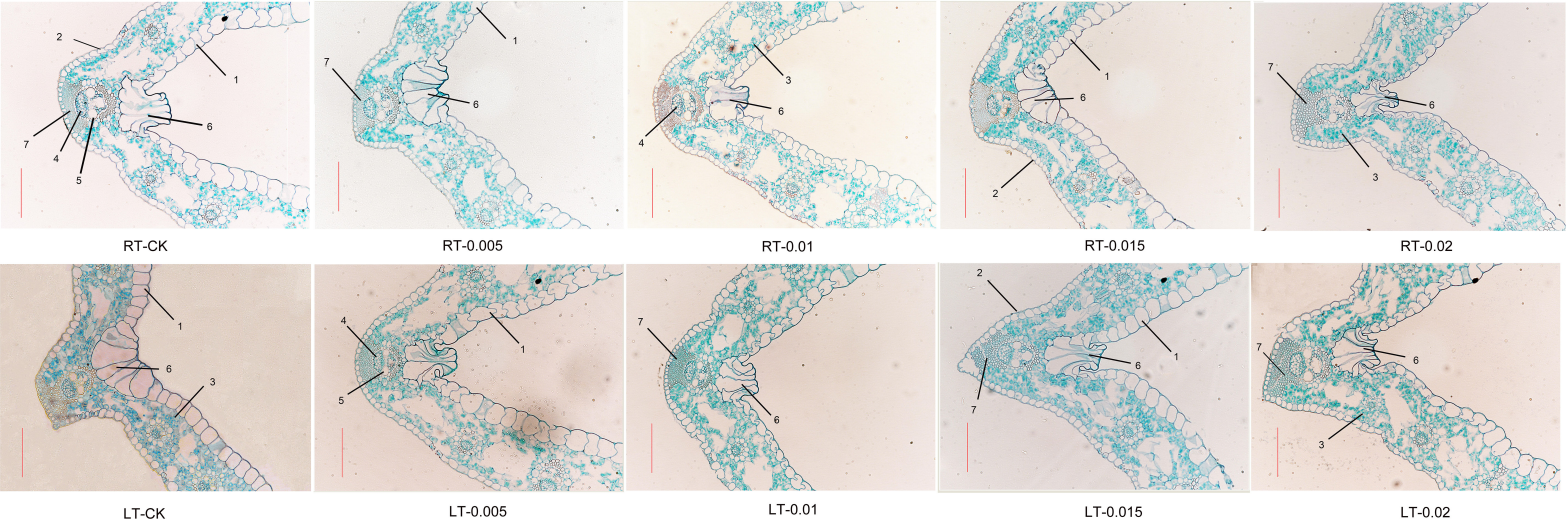
Effect of chitosan on the mesophyll structure of *Kobresia pygmaea* leaves. 1, Upper epidermis; 2, lower epidermis; 3, mesophyll; 4, phloem; 5, xylem; 6, motor cell; 7, mechanical tissue. Scale bars =100 μm.

**Figure 6:**
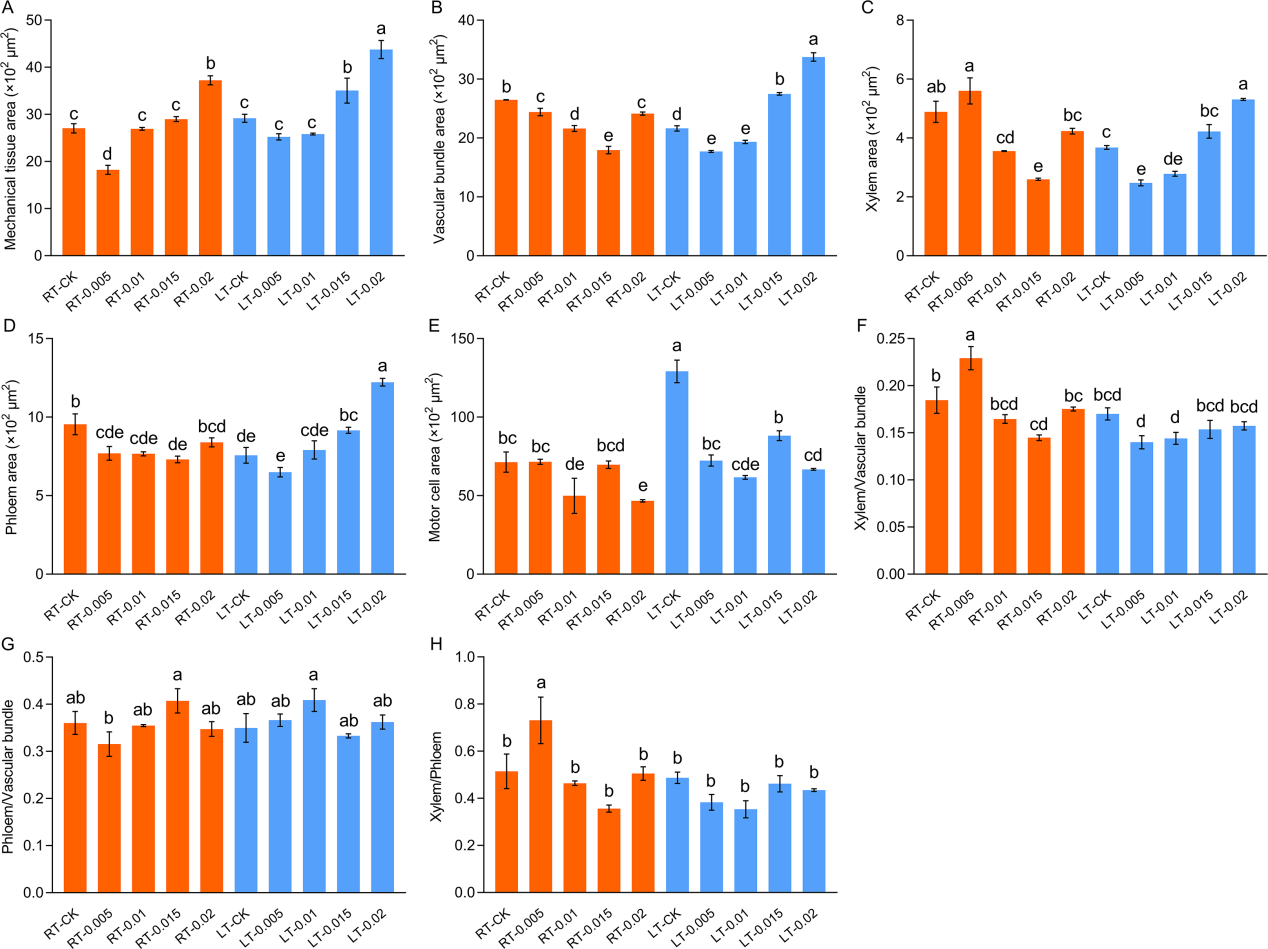
Effect of chitosan on the mesophyll structure, mechanical tissue area **(A)**, vascular bundle area **(B)**, xylem area **(C)**, phloem area **(D)**, motor cell area **(E)**, ratio of xylem/vascular bundle **(F)**, ratio of phloem/vascular bundle **(G)**, and xylem/phloem **(H)** of *Kobresia pygmaea* leaves. Data are represented as mean ± SD (n=3). Different letters above the vertical bars indicate significant differences at a *p* < 0.05 threshold according to Tukey’s range test.

As shown in **Figure 5**, cold stress led to a tight arrangement of the mesophyll and mechanical tissue, and the motor cells expanded and reduced the development of vascular bundles, xylem, and phloem (**Figure 6**). Under cold stress, treatment with 0.015% exogenous CTS notably improved the mesophyll density and thickness of mechanical tissue, induced significant shrinkage of the motor cells, and markedly enhanced the development of vascular bundles.

**Figure 7:**
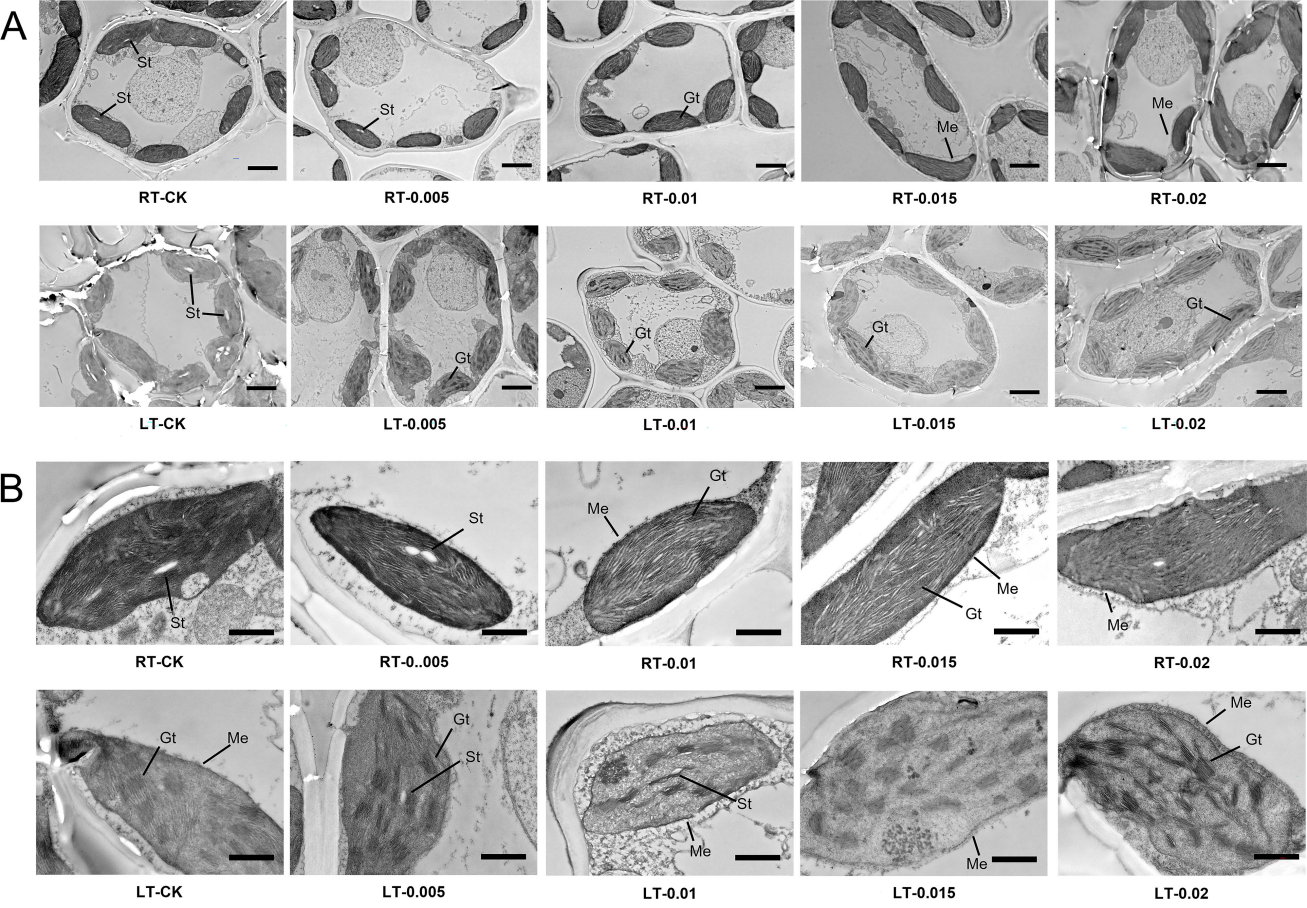
Effect of chitosan on the chloroplast ultrastructure of *Kobresia pygmaea* leaves. **(A)** Whole mesophyll cells; **(B)** magnified view of chloroplast in mesophyll cells. Gt, grana thylakoids; St, starch grain; Me, membrane envelope. Bars in **(A)** were 5 μm; bars in **(B)** were 1 μm.

Under regular temperatures, all the treatments had well-organized stromal thylakoids with typical spindle-shaped chloroplast membrane arrangements. However, cold stress induced structural disruptions, including the swelling of chloroplasts and disorganization of the thylakoid grana lamellae (**Figure 7**). Under cold stress, treatment with exogenous CTS improved the integrity of the cell membrane, which restored the number and orderly arrangement of the thylakoid grana lamellae. The LT-0.015 treatment was particularly effective at inducing this response. Nevertheless, the CTS treatment led to a reduction in the numbers of starch grains, and treatment with 0.02% CTS caused readily apparent damage to the cell membrane under both temperature conditions.

### 3.6 Effect of chitosan on ROS characteristics under cold stress

**Figure 8:**
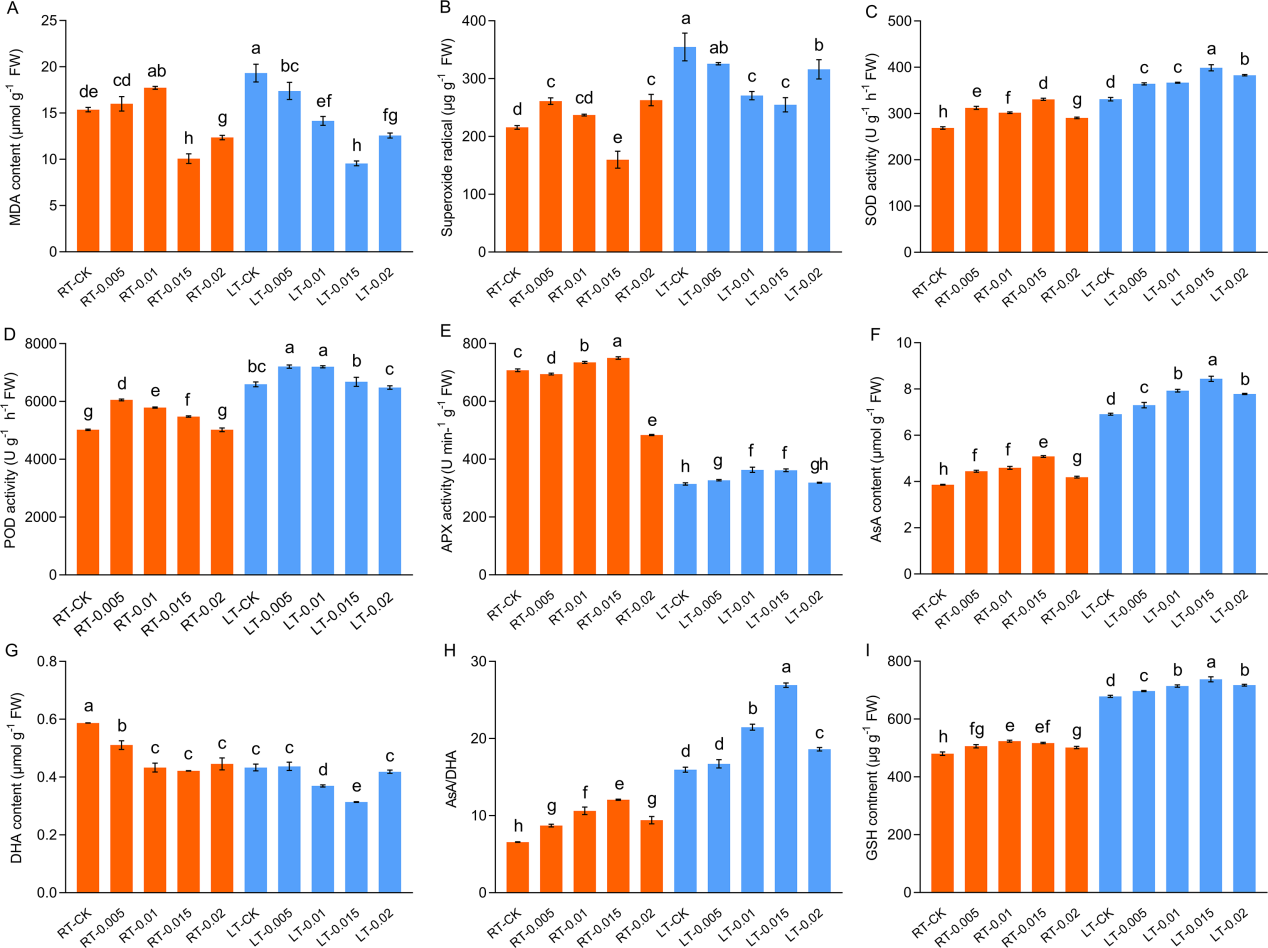
Effect of chitosan on the MDA content **(A)**, superoxide radical **(B)**, SOD activity **(C)**, POD activity **(D)**, APX activity **(E)**, AsA content **(F)**, DHA content **(G)**, AsA/DHA **(H)**, and GSH content **(I)** of *Kobresia pygmaea* leaves. Data are represented as mean ± SD (n=3). Different letters above the vertical bars indicate significant differences at a *p* < 0.05 threshold according to Tukey’s range test.

Previous studies have established that cold stress results in an accumulation of ROS in plants, which triggers oxidative stress. Consistent with this, this study observed a significant increase in the levels of MDA and O_2_•^−^ in the plants subjected to cold stress (LT-CK) compared to the RT-CK group. The application of CTS provided a noticeable protective effect, particularly under cold conditions. Under cold stress, the accumulation of MDA and O_2_•^−^ decreased as the concentrations of CTS increased and reached optimal levels of reduction at a concentration of 0.015%. Furthermore, the levels of MDA and O_2_•^−^ decreased significantly by 50.51% and 28.19%, respectively (**Figure 8**).

Cold stress activated the antioxidant enzyme and non-enzymatic antioxidant systems. Our findings indicated that cold stress led to a significant enhancement in the activities of SOD and POD by 23.18% and 31.33%, respectively, compared with the RT-CK group (**Figure 8**). Furthermore, at different temperatures, the application of 0.015% CTS increased the activities of SOD by 23.08% and 20.55%, compared to the untreated controls. Similarly, at different temperatures, treatment with 0.005% CTS significantly increased the activity of POD by 20.59% and 9.33%, compared to the untreated controls. Conversely, cold stress severely inhibited the activity of APX. In contrast, exogenous treatment with 0.015% CTS significantly markedly restored APX activity, which resulted in an increase of 15.04% compared to the cold treatment.

Cold-stressed plants produce antioxidants to alleviate oxidative damage. This study observed a significant increase of 79.03% in the content of AsA in response to cold stress (**Figure 8**). This coincided with a decrease in the contents of DHA. Compared to the controls at different temperatures, the application of 0.015% CTS was notably effective in enhancing the content of AsA and increased it by 31.72% and 22.19%, whereas the content of DHA was reduced by 28.19% and 27.63%, respectively. The AsA/DHA ratio correlated positively with the changes in levels of AsA, which reflect a coordinated antioxidant response to the stress induced by cold.

### 3.7 Effect of chitosan on chitinase, β-1,3-GA, and PAL activity

**Figure 9:**
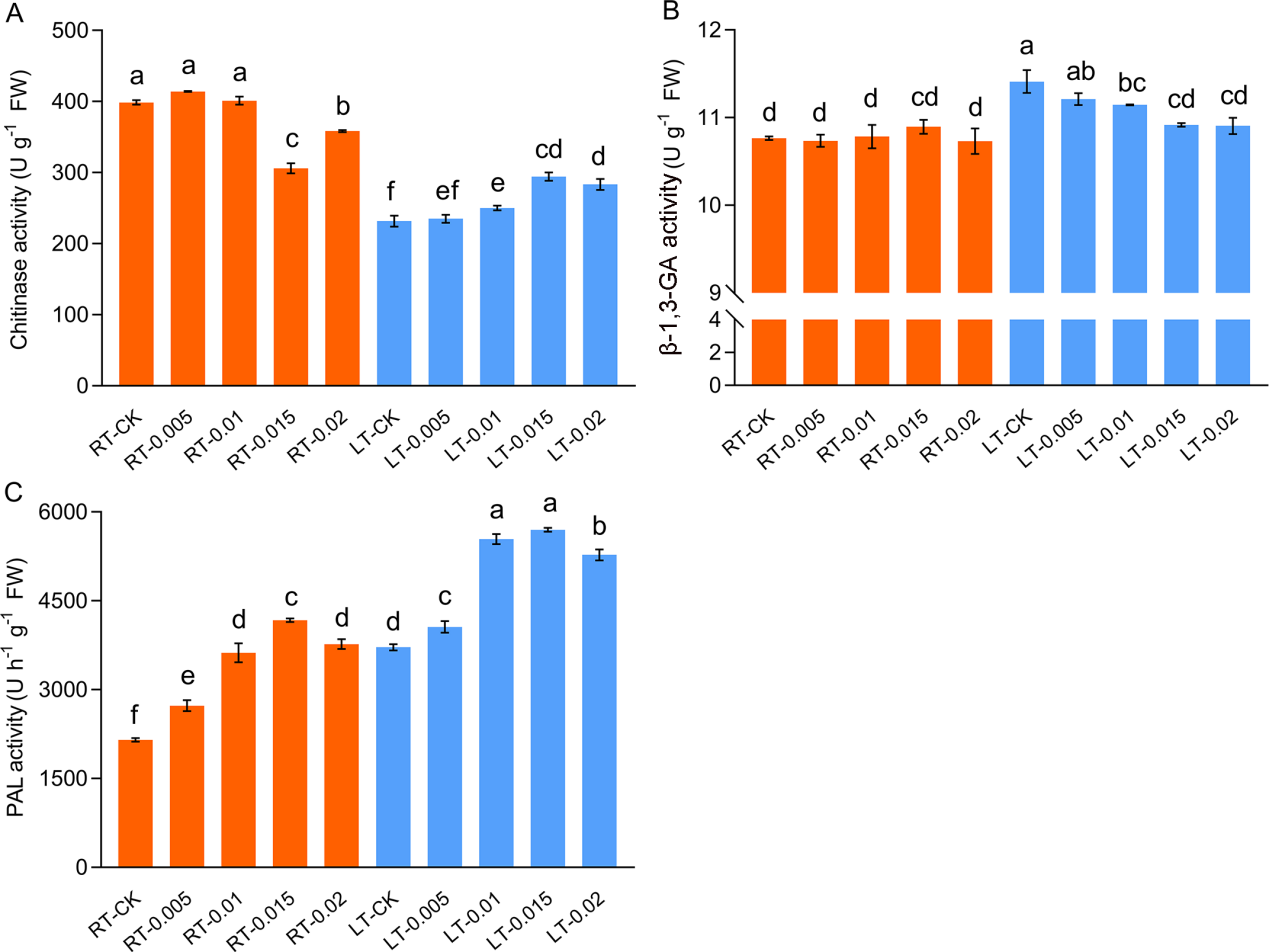
Effect of chitosan on the chitinase **(A)**, β-1,3-GA **(B)**, and PAL **(C)** activity of *Kobresia pygmaea* leaves. Data are represented as mean ± SD (n=3). Different letters above the vertical bars indicate significant differences at a *p* < 0.05 threshold according to Tukey’s range test.

Cold stress differentially affected the activities of chitinase and β-1,3-GA. The activity of chitinase was significantly suppressed by cold stress, whereas the exogenous application of 0.015% CTS enhanced the activity of chitinase by 26.99% compared to the LT-CK group (**Figure 9**). In contrast, the activity of β-1,3-GA was upregulated by cold stress, and this effect was mitigated by the application of CTS, which reduced the activities of β-1,3-GA. The activity of PAL was induced by both cold stress and CTS (**Figure 9**). Compared to the RT-CK group, the activity of PAL increased by 94.02% in the RT-0.015 group and by 72.78% in the LT-CK group. The regulatory effect of CTS on PAL under cold stress mirrored that of chitinase, with a significant 53.33% increase in the LT-0.015% treatment compared to the LT-CK group.

### 3.8 Expression of genes responding to cold stress

**Figure 10:**
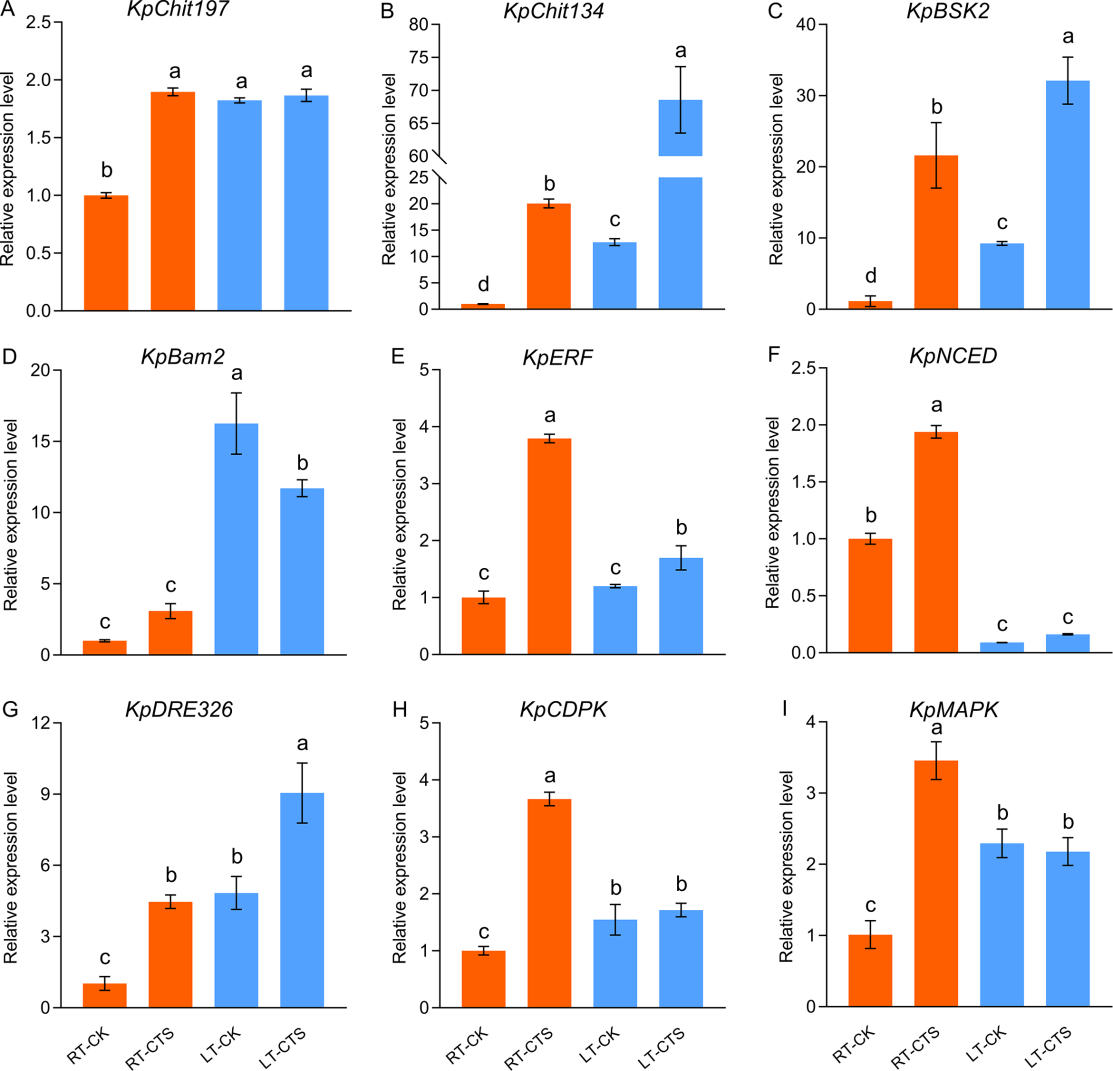
Effect of chitosan on the expression levels of *KpChit197* **(A)**, *KpChit134* **(B)**, *KpBSK2* **(C)**, *KpBam2* **(D)**, *KpERF* **(E)**, *KpNCED* **(F)**, *KpDRE326* **(G)**, *KpCDPK* **(H)**, and *KpMAPK* **(I)** in *Kobresia pygmaea* under cold stress. The expression level of these genes was calculated relative to the control treatment (CK). Data are represented as mean ± SD (n=3). Different letters above the vertical bars indicate significant differences at a *p* < 0.05 threshold according to Tukey’s range test.

We investigated the levels of expression of the genes associated with cold stress tolerance (**Figure 10**). Cold stress significantly upregulated the levels of expression of *KpChit197*, *KpChit134*, *KpBSK2*, *KpBam2*, *KpDRE326*, *KpCDPK*, and *KpMAPK*. Conversely, the expression of *KpNCED*, which is implicated in the biosynthesis of abscisic acid (ABA), was downregulated. The application of CTS further enhanced the levels of expression of *Chit134*, *BSK2*, *ERF*, *NCED*, and *DRE326*.

## 4 Discussion

Cold stress severely disrupts cellular homeostasis, which results in electrolyte leakage and ROS production. This profoundly impairs plant growth and development (Shi Y et al., 2012, Shi H et al., 2014; Varshney et al., 2011). CTS, known for its non-toxic and biodegradable properties, is widely used as a molecular elicitor in agriculture to enhance crop production. In forage crops, CTS has been shown to improve the quality of silage and increase drought tolerance by modulating photosynthesis and energy metabolism (Gandra et al., 2018; Mustafa et al., 2022). Alpine species, such as *K. pygmaea*, have evolved specific adaptations to withstand prolonged cold periods. This study revealed that cold stress inhibited the growth of seedlings, as shown by their slow growth rate, SLA, fresh weight, and weak strength of the sheaths in their stems (**Figure 1**). The morphological changes observed in cold-stressed *K. pygmaea* seedlings, such as withered leaf tips and curled leaves, resembled those documented in previous studies on maize (*Zea mays*) and rice (*Oryza sativa*) (Cofer et al., 2018; Liu H et al., 2022). However, spraying exogenous CTS on cold-stressed seedlings resulted in increased plant height and SLA. This indicates that CTS can promote seedling growth and enhance photosynthetic energy capture, thereby improving cold tolerance.

Cold stress suppressed the metabolic activity of the plants, which reduced their assimilation of nutrients. Li et al. (2011) identified low temperature and nutrients scarcity as primary constraints on the performance of alpine plants. Nitrogenous fertilizers, such as urea and sodium nitroprusside (SNP), are crucial for enhancing the uptake of N, P, and other elements (Sardar et al., 2023; Selvarajh and Ch’ng, 2021). El Amerany et al. (2020) demonstrated that CTS, as a nitrogen-rich organic nitrogen source, improved the acquisition of nutrients, including N, Fe, and Mg, in tomato (*Solanum lycopersicum*) plants grown in infertile soil. Fe and Mg, essential for the biosynthesis of chlorophyll, were quantified to explore their link with chlorophyll accumulation (Tran et al., 2023). In this study, we found that exogenous CTS elevated the accumulation of N and synergistically enhanced the uptake of K, Mg, Fe, and P under cold stress (**Figure 2**). The increase in the levels of Fe and Mg correlated with an increase in chlorophyll. Thus, CTS enhances the uptake of nutrients in cold-stressed plants, which helps them to sustain their growth.

It is notable that CTS exhibits concentration-dependent effects and environmental specificity. Under regular temperature, CTS has no effect on growth but exerts a significant regulatory effect on growth under cold stress. Typically, cold stress leads to the loosening of the mesophyll, which was attributed to water loss and the shrinkage of cell membranes (Yadav, 2010). In this study, we observed tightly arranged mesophyll and mechanical tissues along with expanded motor cells in response to cold stress (**Figure 5**). Treatment with exogenous CTS accelerated the development of mesophyll tissue and the formation of xylem and phloem, which potentially improved water uptake and nutrient transport in stressed seedlings.

Higher plants utilize various protective mechanisms to mitigate the damage induced by stress. The accumulations of diverse osmoprotectants, such as soluble protein, carbohydrates, and glycine betaine, play crucial roles in maintaining the cellular osmotic balance (Cao et al., 2017; Yadav, 2010). In addition to the osmoprotective role of carbohydrates, they provide the energy and substrates necessary for plant metabolic processes. Cold acclimation enhanced the accumulation of carbohydrates in creeping bentgrass (*Agrostis stolonifera*) and annual bluegrass (*Poa annua*) seedlings, but this increase is transient and declines with extended periods of deacclimation (Hoffman et al., 2014). Postharvest applications of CTS suppress transpiration and moisture loss and reduce the conversion of starch and sucrose to fructose and glucose (Zhang et al., 2022). Starch, a key sugar reservoir, is converted to alternative carbohydrates as a cold stress response (Zhang Q et al., 2021). Carbohydrate dynamics, notably the conversion of starch to soluble sugars, is a key mechanism of cold adaptation (Dong and Beckles, 2019; Zhang et al., 2022). CTS has been shown to enhance the metabolism of carbon and increase the content of soluble sugar in tea (*Camellia sinensis*) plants, thereby improving their resistance to cold (Li et al., 2020). In this study, cold stress led to a reduction in starch and an increase in fructose and sucrose. The application of exogenous CTS promoted the degradation of starch and sucrose, which improved the accumulation of fructose (**Figure 2**). Collectively, these data indicate that optimal concentrations of CTS enhance the metabolism of carbohydrates, which bolsters their resistance to cold stress.

Cold stress impairs photosynthesis by inducing damage to leaves and disrupting key photosynthetic processes, including chlorophyll degradation, PSII dysfunction, and diminished photosynthetic enzyme activity (Adhikari et al., 2022). This study found that cold stress markedly reduced the photosynthetic parameters *Pn*, *Gs*, *Tr*, and *WUE*; reduced the levels of chlorophylls; and impaired the activity of Rubisco and efficiency of PSII (**Figure 4**). Chlorophyll is essential to capture energy from light and transport it in photosynthesis, and its reduction leads to diminished light absorption and photosynthetic rates and PSII inefficiency (Demmig-Adams and Adams, 2006; Reinsberg et al., 2001). Exogenous CTS mitigated the degradation of chlorophyll under cold stress, and concurrently, the application of CTS increased the gas exchange indicators (*Pn*, *Tr*, and *Gs*) in cold-stressed seedlings.

Cold stress induces morphological and anatomical changes in the stomata and stems, which leads to the closure of stomata and a decrease in the photosynthetic capacity (Hajihashemi et al., 2018). Stomatal closure is an adaptive mechanism to conserve the water potential and restrict water loss during cold stress (Kozlowski and Pallardy, 1979). Ribulose 1,5-bisphosphate carboxylase/oxygenase (Rubisco), the enzyme central to the fixation of CO_2_ in the Calvin cycle, plays a crucial role in photosynthesis. Enhanced Rubisco activity significantly enhances the photosynthetic capacity (Pignon et al., 2019). A variety of barley (*Hordeum vulgare*) that was more tolerant to cold had a higher degree of stomatal conductance and Rubisco activity when exposed to cold stress (Jurczyk et al., 2018). In this study, the application of CTS at concentrations that ranged from 0.005% to 0.015% under cold stress enhanced the accumulation of chlorophyll and preserved the *Gs* while increasing the activity of Rubisco, thereby improving the photosynthetic capacity of seedlings (**Figure 4**). This suggests that there is a concentration-dependent effect of CTS on *Gs* and Rubisco activity in response to cold stress. CTS has been identified as an anti-transpirant that modulates the biosynthesis of ABA, which leads to the closure of stomata and potentially affects the carboxylation efficiency (Iriti et al., 2009). Under regular temperature, *Gs* and Rubisco activity were inhibited by 0.005% CTS. Conversely, the application of 0.015% CTS improved the *Pn* and *Gs* without enhancing the activity of Rubisco under ambient condition. Otherwise, this concentration also increased the fresh weight and decreased the SLA (**Figure 1**). These findings highlight the intriguing effects of CTS under non-stress conditions and merit further research. Collectively, our results suggest that optimal concentrations of CTS promote photosynthesis and cold tolerance in *K. pygmaea* by enhancing the accumulation of chlorophyll and modulating the Rubisco activity and *Gs* under cold stress.

The chlorophyll fluorescence ratio *Fv/Fm* is a key indicator of PSII (PSII) efficiency under cold stress in plants (Kalaji et al., 2014). In maize, *Fv/Fm*, ETR, and φ*_PSII_* significantly decreased under cold and drought, which was correlated with the degradation of the D1 (PsbA) protein in PSII (Guo et al., 2021). This study observed a similar negative impact of cold stress on the parameters of chlorophyll fluorescence. Cold stress inhibited the electron transport rate (ETR). This led to the accumulation of excess energy in photosystem I (PSI) and the subsequent production of reactive oxygen species (ROS) and photoinhibition. Enhanced non-photochemical quenching (NPQ) serves as a mechanism for the dissipation of excess energy in PSII, thereby alleviating the photoinhibition of PSII caused by cold stress (Da et al., 2018; Porcel et al., 2015). Y(NPQ) reflects the ability of PSII to convert excess excitation energy into heat through the dissipation of regulatory energy (xanthophyll cycle-related energy dissipation). Herein, exogenous CTS mitigated the reduction of *Fv/Fm*, φ*_PSII_*, and the ETR induced by cold stress, while it promoted Y(NPQ). Additionally, the cold-stressed seedlings treated with CTS exhibited a significant reduction in Y(NO) compared to the LT-CK group (**Table 2**). Y(NO) represents the non-regulatory energy dissipation of PSII and refers to the ability of PSII to passively dissipate excitation energy by closing the reaction center of PSII owning to photoinhibition damage. It also serves as an indicator for the photooxidative damage inflicted upon PSII (Liu et al., 2012). Additionally, carotenoids, such as lutein, zeaxanthin, violaxanthin, and neoxanthin, are integral to the xanthophyll cycle and play vital roles in the dissipation of heat during the process of photoinhibition under intense light or UV-B radiation (Demmig-Adams and Adams, 2006; Pereira et al., 2021; Reinsberg et al., 2001). In this study, the production of carotenoids was induced in response to cold stress and further enhanced by the application of CTS, which contributed to the protection of photosystem under cold stress. These findings demonstrate that CTS can enhance resistance against cold stress by regulating the distribution of energy within the reaction center of PS II.

Chloroplasts serve as sites for photosynthetic reactions and act as pivotal sensors and responders to low temperatures (Crosatti et al., 2013; Gao et al., 2022). This study found that cold stress inflicted severe cellular damage, including the disruption of chloroplast membranes and the organization of thylakoid grana lamellae (**Figure 6**). Previous studies have reported that low temperature impacts chloroplasts by inducing the swelling of the thylakoids and chloroplasts, while inhibiting enzymatic reactions on the thylakoid membrane (Zhuang et al., 2019). In plants resistant to chilling, the starch granules gradually diminish over time and are associated with an increased density of grana disks (Garstka et al., 2007; Kratsch and Wise, 2000; Zhuang et al., 2019). We observed a reduction in starch granules in the seedlings stressed by cold, with the grana lamellae appearing loosely arranged (**Figure 7**). The application of CTS under cold stress induced a typical spindle-shaped vesicle-like chloroplast membrane arrangement and increased the number of grana thylakoids (Gt), which are essential for chlorophyll attachment and light reactions. CTS effectively preserved membrane integrity and mitigated the chloroplast damage caused by cold stress.

Cold stress severely disrupts photosynthesis in the chloroplasts, which results in the generation of ROS by surplus photo-energy. This leads to membrane damage, the denaturation of biomolecules, and cell death (Alam and Jacob, 2002; Mittler, 2017). To combat this oxidative stress, plants activate their antioxidant defense systems, which include both enzymatic antioxidants such as SOD, glutathione peroxidase (GPX), and POD, and nonenzymatic antioxidants, such as AsA (Faize et al., 2011; Rastegari et al., 2022; Yu et al., 2022). This study found that cold stress significantly increased the production of MDA and O_2_•^−^ and also significantly enhanced the activities of POD and SOD (**Figure 8**). The application of 0.015% exogenous CTS was particularly effective at reducing the levels of MDA and O_2_•^−^ and enhancing the activities of SOD, POD, and APX under cold stress (**Figure 8**). Our findings corroborate previous research that has shown that CTS boosts antioxidant enzyme activity (Nie et al., 2020). Notably, the levels of nonenzymatic antioxidants AsA and GSH increased under cold stress, and the CTS treatment significantly enhanced this response. We propose that CTS modulates the antioxidant defenses by regulating both enzymatic activities, such as SOD, POD, and APX, and nonenzymatic components, such as AsA and GSH. As a dominant species on the Tibetan Plateau, the high antioxidant capacity in *K. pygmaea* may be pivotal for its cold resistance, thus suggesting a promising area for future research.

CTS activates the phytoalexins in host cells and triggers secondary metabolites. It has been shown to enhance the activity of chitinase-like proteins (CTLs), which are involved in the biosynthesis of cell walls and contribute to the differentiation of cells and the development of plants in a manner similar to that of cellulose synthases (Hermans et al., 2010; Sanchez-Rodriguez et al., 2012). β-1,3-GA is responsible for the degradation of callose in plants. Chitinase and β-1,3-GA were induced to increase during the process of the promotion of the germination of pepper (*Capsicum annuum*) seeds by CTS (Samarah et al., 2020). Our research showed that cold stress initially suppressed chitinase and stimulated β-1,3-GA activity (**Figure 9**). However, the application of CTS reversed this trend. This modulation by CTS might be presumed to enhance the biosynthesis of cell walls and the deposition of callose. Previous studies have demonstrated that elicitor CTS promoted the productions of phenolics and flavonoids compounds, which are the productions catalyzed by PAL, thus effectively enhancing the antioxidant capacity of tissues (Khan et al., 2019; Park et al., 2019). Consistent with these findings, our results indicate that CTS treatment under cold stress substantially upregulated the activity of PAL and acted synergistically to regulate cold tolerance in *K. pygmaea* (**Figure 9**).

In *K. littledalei*, cold stress induces the expression of genes homologous to *BAM3_ARATH* and *BSK5_ARATH*, which suggests that they are involved in stress response pathways (Qu et al., 2021). BAM2, a receptor kinase, has been implicated in the perception of CLE peptides, which are essential for plant development, including root growth and patterning (Hu et al., 2022). Brassinosteroids signaling kinases (BSKs) are substrates of BR kinases, which activate downstream BR signaling pathways under stress conditions, thereby mediating the BR signaling pathway (Tang et al., 2008). Under cold stress, the levels of expression of *KpBAM* and *KpBSK2* were upregulated, and CTS further enhanced the expression of *KpBSK2*. This modulation, along with the altered expression of genes involved in hormone metabolism and stress response pathways—such as C-repeat binding factor/dehydration-responsive element-binding factor 1, the MAPK cascade, and CDPKs—implicates their role in enhancing cold resistance and growth in plants (Adhikari et al., 2022; Miao et al., 2023). In this study, exogenous CTS induced the expression of additional genes, including *Chit134*, *BSK2*, *ERF*, *NCED*, and *DRE326* (**Figure 10**). We propose that these genes are integral to the biosynthesis of dehydrin proteins and the regulation of hormone metabolism, which collectively contribute to resistance to cold stress. This hypothesis is bolstered by the observed upregulation of these genes in response to both cold stress and CTS treatment.

## 5 Conclusion

Cold stress inhibited the growth of *Kobresia pygmaea* and caused photoinhibition and oxidative damage. However, treatment with exogenous chitosan enhanced its resistance to cold by increasing the uptake of nutrients; optimizing the metabolism of carbohydrates; increasing the activities of enzymes, such as SOD and APX, and the levels of antioxidants, such as AsA and GSH; and maintaining photosynthesis and the activity of enzymes, such as Rubisco. CTS also promoted the development of vascular bundles, the stability of chloroplast structure, and the induction of chitinase and PAL enzymes, along with upregulating the cold-responsive genes. This treatment also promoted the expression of genes related to cold tolerance. Collectively, these physiological and biochemical changes induced by CTS suggest its potential in enhancing the cold tolerance and growth of *K. pygmaea* seedlings, thus offering insights for the application of CTS in the cultivation of alpine plants.

## Data Availability

The original contributions presented in the study are included in the article/**Supplementary Material**. Further inquiries can be directed to the corresponding authors.

## Supplementary material

Supplementary Table 1 The primers for qRT-PCR.

Supplementary Figure 1 Principal component analysis (PCA) showing score plots discriminating the different temperatures with or without chitosan treatments by virtue of the first 2PCs, where attributes in A is represented as: 1: Plant height, 2: SLA, 3: Fresh weight, 4: Total chlorophyll, 5: Chl *a*, 6:Chl *b*, 7: Car, 8: Chl *a/b*, 9: *Pn*, 10: *Gs*, 11: *Ci*, 12: *Tr*, 13: *WUE*, 14: *Fv/Fm*, 15: φ*_PSII_*, 16: ETR, 17: YNO, 18: YNPQ, 19: Rubisco, 20: MDA, 21: O_2_^-^, 22: POD, 23: SOD, 24: APX, 25: AsA, 26: DHA, 27: GSH, 28: Chitinase, 29: β-1,3-GA, 30: PAL, 31: Soluble sugar, 32: Soluble protein, 33: Fructose, 34: Starch, 35: Sucrose, 36: N, 37: P, 38: K, 39: Fe, 40: Mg, 41: Sugar dry mass, 42: Ca dry mass, 43: Crude protein dry mass, 44: Fiber dry mass.

Supplementary Figure 2 Pearson’s correlation analysis among physiochemical attributes after exogenous chitosan application under cold stress. Red color represents positive correlation and blue color represents negative correlation according to the correlation coefficient (from +1.0 to −1.0). One, two or three asterisks indicate significance corresponding to *P* < 0.05, 0.01 or 0.001 (correlation coefficient, Pearson).

Supplementary Figure 3 Effect of chitosan on near-infrared indicators of dry matter, sugar dry matter **(A)**, calcium dry matter **(B)**, crude protein **(C)**, fiber dry matter **(D)**, neutral detergent fiber dry matter **(E)**, acid detergent fiber **(F)** of *Kobresia pygmaea* leaves. Data were represented as mean ± SD (n=4). Different letters above the vertical bars indicate significant differences at a *P* < 0.05 threshold according to Tukey’s range test.

Supplementary Figure 4 Effect of chitosan on Chl *a/b* of *Kobresia pygmaea* leaves. Data were represented as mean ± SD (n=3). Different letters above the vertical bars indicate significant differences at a *P* < 0.05 threshold according to Tukey’s range test.
