# Supplementary material for: Chitosan induced cold tolerance in Kobresia pygmaea by regulating photosynthesis, antioxidant performance, and chloroplast ultrastructure
Source: Front Plant Sci. 2024 Nov 20;15:1441564. doi: 10.3389/fpls.2024.1441564 (PMC11614638; doi:10.3389/fpls.2024.1441564)
Supplement: Supplementary Figure 1 — Principal component analysis (PCA) showing score plots discriminating the different temperatures with or without chitosan treatments by virtue of the first 2PCs, where attributes in A is represented as: 1: Plant height, 2: SLA, 3: Fresh weight, 4: Total chlorophyll, 5: Chl a, 6:Chl b, 7: Car, 8: Chl a/b, 9: Pn, 10: Gs, 11: Ci, 12: Tr, 13: WUE, 14: Fv/Fm, 15: φ PSII , 16: ETR, 17: YNO, 18: YNPQ, 19: Rubisco, 20: MDA, 21: O2 -, 22: POD, 23: SOD, 24: APX, 25: AsA, 26: DHA, 27: GSH, 28: Chitinase, 29: β-1,3-GA, 30: PAL, 31: Soluble sugar, 32: Soluble protein, 33: Fructose, 34: Starch, 35: Sucrose, 36: N, 37: P, 38: K, 39: Fe, 40: Mg, 41: Sugar dry mass, 42: Ca dry mass, 43: Crude protein dry mass, 44: Fiber dry mass. [file DataSheet2.docx]

**Supplementary Table 1**

The primers for qRT-PCR.

| Gene name | Forward Primer | Reserve Primer |
| --- | --- | --- |
| *GAPDH* | GGAGGAGTCTGAGGGCAAAC | TGGCGGACTAGGTCAACAAC |
| *KpChit197* | GACCGCACAATCACCAAAAC | TTACCACACTCAATGCCTCC |
| *KpChit134* | TGTGCAGTCATCCAATACCAG | AGTACAACTACGAACAAGCCG |
| *KpBSK2* | CTAGTAAGGGTCGTGCTCTTTATC | GTCGTTCCCTTGGTTCATACT |
| *KpBam2* | CGATGAAGTATGACTGGGAAGG | CGCAACCGAGAGAGATGTATT |
| *KpERF* | ACTGGGATCGAGTCTGACGA | GTGTTGAAGGTACCGAGCCA |
| *KpNCED* | CTTGTTTGACGGTGATGGC | TTTGGACATAACGGGAGCAG |
| *KpDRE326* | TCTTCAACCTCCAACGACTTC | TGACGCACACCTTTGTAGAC |
| *KpCDPK* | AGGTGCGTATGAAGACAAGAG | CCACATTCACTATTGCTCTGC |
| *KpMAPK* | AGTCCTCTTTGCATCCATGTG | CAAGTATCAGCCTCCGATCATG |

**
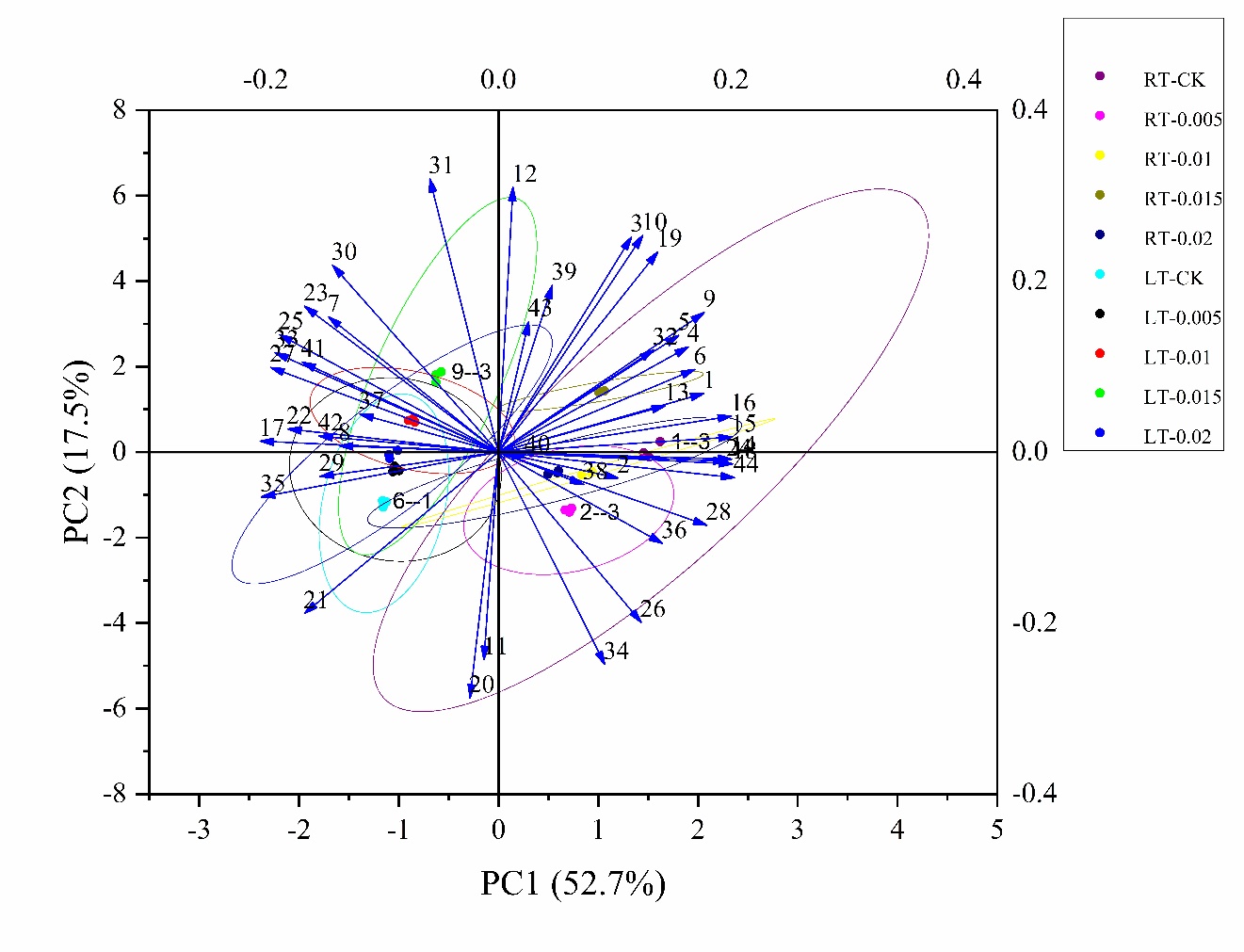
**

**Supplementary Figure 1** Principal component analysis (PCA) showing score plots discriminating the different temperatures with or without chitosan treatments by virtue of the first 2PCs, where attributes in A is represented as: 11: Plant height, 2: SLA, 3: Fresh weight, 4: Total chlorophyll, 5: Chl *a*, 6:Chl *b*, 7: Car, 8: Chl *a/b*, 9: *Pn*, 10: *Gs*, 11: *Ci*, 12: *Tr*, 13: *WUE*, 14: *Fv/Fm*, 15: φ*_PSII_*, 16: ETR, 17: YNO, 18: YNPQ, 19: Rubisco, 20: MDA, 21: O_2_^-^, 22: POD, 23: SOD, 24: APX, 25: AsA, 26: DHA, 27: GSH, 28: Chitinase, 29: β-1,3-GA, 30: PAL, 31: Soluble sugar, 32: Soluble protein, 33: Fructose, 34: Starch, 35: Sucrose, 36: N, 37: P, 38: K, 39: Fe, 40: Mg, 41: Sugar dry mass, 42: Ca dry mass, 43: Crude protein dry mass, 44: Fiber dry mass.





**Supplementary Figure 2** Pearson’s correlation analysis among physiochemical attributes after exogenous chitosan application under cold stress. Red color represents positive correlation and blue color represents negative correlation according to the correlation coefficient (from +1.0 to −1.0). One, two or three asterisks indicate significance corresponding to *P* < 0.05, 0.01 or 0.001 (correlation coefficient, Pearson).


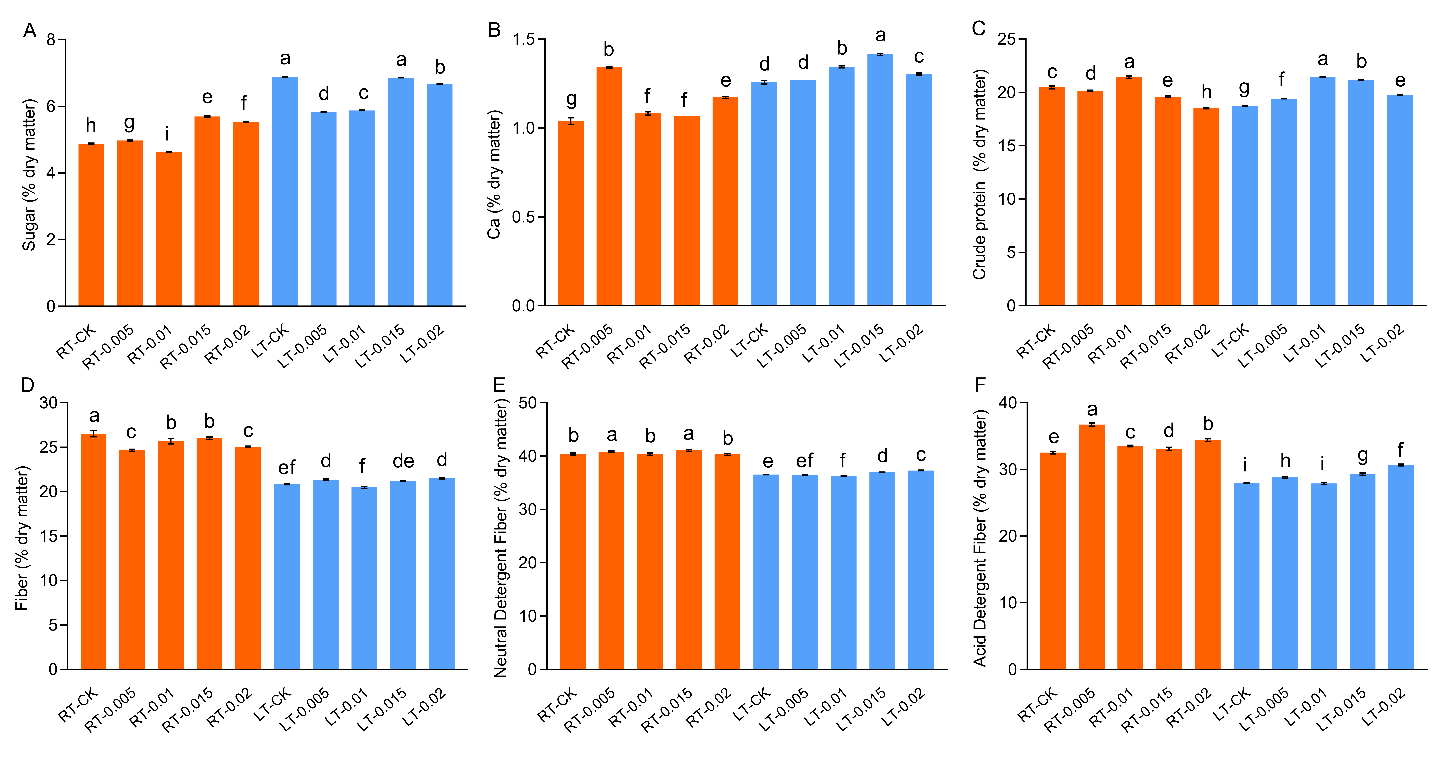


**Supplementary Figure 3.** Effect of chitosan solutions on the near-infrared indicators of dry matter, sugar dry matter (A), calcium dry matter (B), crude protein (C), fiber dry matter (D), neutral detergent fiber dry matter (E), acid detergent fiber (F) of *Kobresia pygmaea* leaves. Data were represented as mean ± SD (n=4). Different letters above the vertical bars indicate significant differences at a *P* < 0.05 threshold according to Turkey’s range test.


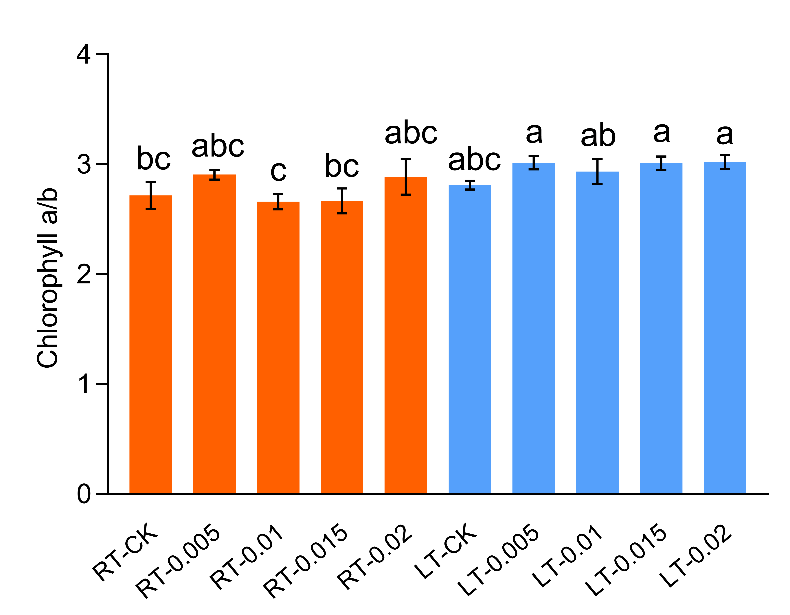


**Supplementary Figure 4** Effect of chitosan on Chl *a/b* of *Kobresia pygmaea* leaves. Data were represented as mean ± SD (n=3). Different letters above the vertical bars indicate significant differences at a *P* < 0.05 threshold according to Turkey’s range test.
